# Extranuclear DNA accumulates in aged cells and contributes to senescence and inflammation

**DOI:** 10.1111/acel.12901

**Published:** 2019-01-31

**Authors:** Yuk Yuen Lan, James M. Heather, Thomas Eisenhaure, Christopher Stuart Garris, David Lieb, Raktima Raychowdhury, Nir Hacohen

**Affiliations:** ^1^ Center for Cancer Research Massachusetts General Hospital Charlestown Massachusetts; ^2^ Broad Institute Cambridge Massachusetts; ^3^ Department of Medicine Harvard Medical School Boston Massachusetts; ^4^ Center for Systems Biology Massachusetts General Hospital Boston Massachusetts; ^5^ Graduate Program in Immunology Harvard Medical School Boston Massachusetts

**Keywords:** cellular senescence, Dnase2a, extranuclear DNA, inflammation, premature aging, STING pathway

## Abstract

Systemic inflammation is central to aging‐related conditions. However, the intrinsic factors that induce inflammation are not well understood. We previously identified a cell‐autonomous pathway through which damaged nuclear DNA is trafficked to the cytosol where it activates innate cytosolic DNA sensors that trigger inflammation. These results led us to hypothesize that DNA released after cumulative damage contributes to persistent inflammation in aging cells through a similar mechanism. Consistent with this notion, we found that older cells harbored higher levels of extranuclear DNA compared to younger cells. Extranuclear DNA was exported by a leptomycin B‐sensitive process, degraded through the autophagosome–lysosomal pathway and triggered innate immune responses through the DNA‐sensing cGAS‐STING pathway. Patient cells from the aging diseases ataxia and progeria also displayed extranuclear DNA accumulation, increased pIRF3 and pTBK1, and STING‐dependent p16 expression. Removing extranuclear DNA in old cells using DNASE2A reduced innate immune responses and senescence‐associated (SA) β‐gal enzyme activity. Cells and tissues of *Dnase2a^−^* ^/^ *^−^* mice with defective DNA degradation exhibited slower growth, higher activity of β‐gal, or increased expression of HP‐1β and p16 proteins, while *Dnase2a^−^* ^/^ *^−^*;*Sting^−^* ^/^ *^−^* cells and tissues were rescued from these phenotypes, supporting a role for extranuclear DNA in senescence. We hypothesize a direct role for excess DNA in aging‐related inflammation and in replicative senescence, and propose DNA degradation as a therapeutic approach to remove intrinsic DNA and revert inflammation associated with aging.

## 1 INTRODUCTION

Aging is associated with increased risk of heart disease, cancer, diabetes, cognitive decline, and many other pathological conditions. While numerous mechanisms are likely to be relevant, systemic changes in the inflammatory response with aging are likely to impact the risk of these diverse conditions. Indeed, subclinical but heightened inflammation is observed in aging tissues, and in the blood of older adults in large epidemiologic studies, with consistently higher basal levels of C‐reactive protein and abundant pro‐inflammatory cytokines like IL‐6, IFN‐β, and TNF‐α (Fagiolo et al., 1993; Roubenoff et al., 2003; Singh & Newman, 2011). Such alteration is often viewed as noncell autonomous, for example senescent cells, which increase with aging, may modulate inflammation through secretion of cytokines (i.e., senescence‐associated secretory phenotype, SASP (Coppé et al., 2010)). The intrinsic processes that initiate this inflammation in aging remain largely unknown**.**

We previously described a cell‐autonomous process in which damaged nuclear DNA is trafficked to the cytosol, transported via autophagy, and degraded by lysosomal nuclease DNASE2A. Excess DNA accumulated under conditions of increased damage, defective degradation, or autophagy blockade can activate the STING pathway leading to inflammation (Lan, Londono, Bouley, Rooney, & Hacohen, 2014). DNA damage has been postulated to be a major cause of cellular aging. We hypothesize that cumulative damage may generate excess DNA leading to persistent inflammation in aging cells through a similar mechanism. Several observations in senescence seem to agree with our prediction. Unrepaired or persistent double‐stranded breaks (DSBs) can be found in senescing cells and aging human and animal tissues (Rube et al., 2011; Sedelnikova et al., 2004; Wang et al., 2009), and cells are known to senesce upon DNA damage (Le et al., 2010). Senescent nuclei also undergo dramatic chromatin changes with fragments budding off the nucleus and being processed into autophagic vesicles (Ivanov et al., 2013). Senescence and aging phenotypes in mice rely on type I IFN signaling (Yu et al., 2015), which is one key response upon STING activation. Recent studies then reveal the involvement of DNA sensors in the induction of senescence (Glück et al., 2017; Yang, Wang, Ren, Chen, & Chen, 2017), and nuclear DNA as the stimulatory ligand for nonautonomous SASP (Dou et al., 2017). Here, we test our original model in the context of aging, observing how nuclear DNA connects the intrinsic processes of damage, autophagy, sensing, degradation, and the induction of innate immune responses.

We used cellular replicative senescence as a model of aging and analyzed effects of extranuclear DNA on inflammation and senescence. In young and old human diploid fibroblasts (based on their replication age/population doubling, PD), we compared their levels of extranuclear DNA, transcriptional profiles, and sensing of intrinsic DNA in modulating innate immune responses. We then extended our study to clinical conditions of premature aging syndromes. We further tested a therapeutic approach of removing autonomous DNA to reduce inflammation in old cells. Finally, using mice deficient in DNASE2A, we observed a role for autonomous DNA and the STING pathway in promoting senescence. Altogether, we demonstrate that excess DNA contributes to inflammation and senescence, and reveal components of the DNA sensing and degradation machinery that could be targeted for modulation of aging‐related innate immune responses.

## 2 RESULTS

### 2.1 Older cells accumulate DNA outside the nucleus

**Figure 1:**
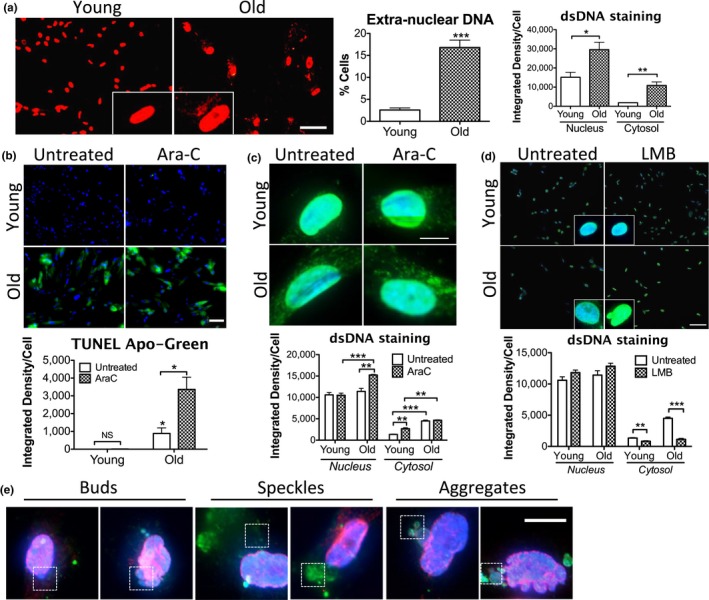
Old cells accumulate nuclear DNA in the cytosol. (a) IF staining of anti‐dsDNA (red) in young and old WI38 cells. Insets, enlarged cells; scale bar, 50 μm. Quantitation shows manual cell count with extranuclear DNA in percentage (middle panel) and signal intensity per cell in nucleus and cytosol (right panel). (b) TUNEL assay (green) detecting DNA fragmentation in young and old MRC5 cells without or with Ara‐C treatment. Scale bar, 50 μm. (c) IF staining and quantitation of anti‐dsDNA (green) in young and old MRC5 cells untreated or treated with Ara‐C. Scale bar, 20 μm. (d) IF staining and quantitation of anti‐dsDNA (green) in young and old MRC5 cells untreated or treated with leptomycin B (LMB, 20 nM, 24 hr). Insets show enlarged cells. Scale bar, 50 μm. (e) Dual staining of dsDNA (green) and NUP98 (red) in old MRC5 cells. Dotted squares highlight DNA patterns indicated; scale bar, 20 μm. Results are representative of 3 (a) or 2 (b, c, d, e) independent experiments. Ara‐C treatment, 10 μM, 24 hr. DAPI (blue) used as counterstain in b–e. Quantitation is based on 5 random fields at 10× or 20× in representative experiment. Values in quantitation are mean ± *SEM*. *p*‐value of significance by *t* test, *p < 0.05, **p < 0.01, ***p < 0.005, ****p < 0.0001

Based on prior studies showing increased DNA damage in older cells and our findings that excess damaged nuclear DNA leads to elevated cytosolic DNA, we hypothesized that cytosolic DNA would be more abundant in old compared to young cells. Using anti‐double‐stranded DNA (dsDNA) antibodies to detect DNA by immunofluorescence (IF), we found that 16.8% of old WI38 cells (human lung fibroblasts at PD68–70, approaching senescence) exhibited extranuclear DNA, in contrast to 2.6% in young cells (PD25–30), and DNA in the cytosol of old cells at much higher intensity than in young cells (Figure 1a). These observations were confirmed in another fibroblast line, MRC5 (Figure 1c, untreated). DNase1 digestion after fixation and permeabilization removed most of the cytosolic signals in old cells (Figure S1A), thus verifying that the signal in the cytosol was due to DNA as we showed previously (Lan et al., 2014).

To confirm the presence of damaged DNA, we used TUNEL staining to label DNA nicks. Nicked DNA was strongly visible in old cells, prominently in the cytosol, but undetectable in young cells (Figure 1b, untreated), and was more intense in old cells upon induction of DNA damage by the DNA damaging agent cytarabine/Ara‐C which causes DSBs (Figure 1b, Ara‐C‐treated). Ara‐C treatment also led to more extranuclear DNA in young cells (mostly speckles) and more nuclear DNA in old cells (Figure 1c), suggesting there may be a saturation of DNA export to the cytosol.

### 2.2 Nuclear origin and export of damaged DNA in aging cells

Excess mitochondrial DNA or mitochondria are not likely to be a source of the excess DNA in old cells since MitoTracker did not co‐localize with excess DNA (Figure S1B), though we do not exclude escape of DNA from mitochondria. However, blocking of nuclear transport to the cytosol with leptomycin B (LMB) led to a dramatic reduction in cytosolic DNA in old cells (Figure 1d), along with some increase in dsDNA staining in nuclei of LMB‐treated cells (likely due to blockage of export). Dual staining of dsDNA and a nuclear envelope marker NUP98 (nucleoporin 98) revealed patterns of nuclear DNA egress in old cells in the form of buds at the nuclear perimeter and speckles and large aggregates in the cytosol (which are patterns observed in our prior study) (Figure 1e). Distribution of NUP98 was uneven or disrupted and nuclear lobulations could be severe (Figure 1e, right panel). Both results support the nuclear origin of extranuclear DNA in these (likely nonphagocytic) fibroblasts.

In our model, autophagy is required for clearance of damaged nuclear DNA. We found an increased expression of autophagy genes in old cells compared with young cells, including *ATG5*, *BECLIN1,* and transcription regulators *P62* and *PTEN* (Figure S1C), and the protein products of autophagosome marker LC3 and lysosomal protein LAMP1 (Figure S1D). Indeed, extranuclear DNA co‐localized with LC3 and LAMP1 in old cells (Figure S1E), representing association of the autophagosome–lysosomal pathway similar to what we previously described. The co‐localization of DNA and LC3 is not consistent with an extracellular source of DNA, such as exosomes or apoptotic cells and debris. We also found a high percentage of SA‐βgal+ cells in aged MRC5 cells that further increased upon induced damaged by Ara‐C, but none in young cells (Figure S1F), consistent with this marker reflecting lysosomal abundance. Supporting these results, inducing autophagy in old cells with rapamycin reduced the amount of cytosolic DNA accumulation (Figure S1G). We conclude that cells of older replicative age have increased levels of extranuclear DNA fragments that are being transported from the nucleus and processed via autophagy.

### 2.3 Innate immune expression profiles in old cells

**Figure 2:**
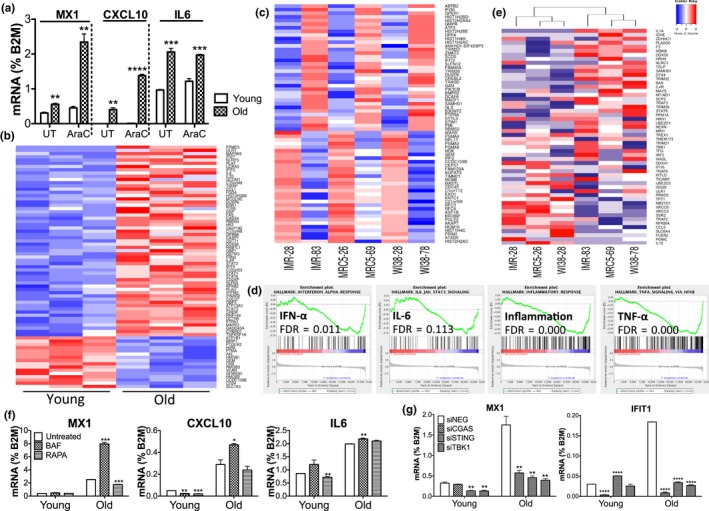
Innate immune activation in old cells. (a) Expression of IFNI‐inducible and inflammatory genes in young and old WI38 cells by RT‐qPCR, UT, untreated, Ara‐C treated, 10 μM, 24 hr. (b) Heat map showing significantly upregulated (top) or downregulated (bottom) genes in old versus young MRC5 cells by NanoString in three biological replicates (full gene list in Figure S2A). (c) Heat map showing DEGs in old cells that overlap with IFNI‐regulated genes restricted to fibroblasts (35 upregulated on top and 31 downregulated genes on bottom, full list in Figure S2B). Numbers after each cell line indicate PD numbers. (d) Enriched GSEA‐ranked gene sets in old compared with young cells across IMR90, MRC5, and WI38 cell lines based on RNA‐seq data. Enrichment in old cells is at low end of ranking, FDR for significance. (e) Unsupervised clustering of young and old cells based on STING‐related genes (full list in Figure S2G). (f) Gene expression of MX1, CXCL10, and IL‐6 in young and old MRC5 cells by RT‐qPCR, untreated or treated with bafilomycin A (BAF, 20 nM) or rapamycin (RAPA, 1 nM) for 8 hr, significance relative to untreated cells. (g) Transcript expression of MX1 and IFIT1 in young and old MRC5 cells measured by RT‐qPCR after knocking down *cGAS*, *STING,* or *TBK1* by transfected siRNAs, significance relative to *siNEG*, nontargeting control. Results in a, f, and g are representative of 2 independent experiments, p‐values of significance by *t* test

Accumulated extranuclear DNA can provoke an increased expression of type I IFN and inflammatory cytokines and genes via the STING pathway. Despite undetectable levels of IFN‐α and IFN‐β (and IFN‐λ) transcripts, we confirmed by RT‐qPCR higher basal levels of type I IFN‐inducible and inflammatory genes MX1, CXCL10, and IL‐6 in old MRC5 cells compared with young cells, which were further increased upon Ara‐C treatment (Figure 2a). This suggests stronger activation of immune responses and higher sensitivity to damage in old than in young cells. To focus on innate immune activation, we measured transcripts of 413 innate and inflammation‐related genes using a custom human NanoString multiplex panel. We observed 59 significantly upregulated genes in old MRC5 cells (Figure 2b), which overlapped with the type I IFN (e.g., IFIT2, IFIT5, IFNAR2, STAT1, STAT2) and IL‐6‐JAK‐STAT3 (e.g., IL‐6, STAT3, STAT6) pathways, and downregulated genes that included HMGB1, 2, and 3 (nonhistone nuclear proteins of the Alarmin family that trigger immune responses) (Figure S2A, full gene list). To examine the aging transcriptome more broadly for essential innate immune components, we performed RNA sequencing (RNA‐seq) of young and old cells from three common human diploid fibroblasts: IMR90 and WI38 together with MRC5. We identified differentially expressed genes (DEGs) in old versus young cells: 683 upregulated and 698 downregulated DEGs. Using a curated set of 625 type I IFN‐regulated genes in fibroblasts (Interferome v2.01; (Rusinova et al., 2013)), we found a significant overlap of 35 upregulated and 31 downregulated DEGs in old cells (Figure 2c; Figure S2B, gene list; Figure S2C, significance *p* < 0.0001 by hypergeometric test; Figure S2D, actual > predicted frequencies by permutation test), with some heterogeneity across cell lines (Figure S2E).

Gene Set Enrichment Analysis (GSEA) revealed that old cells, across all three cell lines, were enriched in genes that are part of the “IFN‐α response,” “IL‐6‐JAK‐STAT3 signaling,” “inflammatory response,” and “TNF‐α signaling” (Figure 2d; Figure S2F, other hallmarks with False Discovery Rate FDR < 0.25)—each Hallmark gene set is minimally redundant to represent the denoted pathway. IFN response and IL‐6 represent the two arms of inflammatory responses downstream of DNA sensing (TBK1‐IRF3 axis and IKK‐NF‐κB axis, respectively (Li & Chen, 2018)). Using only 54 STING‐interacting factors (Figure S2G, pathwaycommons.org), unsupervised hierarchical clustering separated young and old cells, with 15% of these genes significantly upregulated in old cells including IL1A, F3, IKBKB, TSLP, SAMHD1, DTX4, DDX41, and IL4R (Figure 2e).

### 2.4 Role of autophagy and sensing in old cell innate immune activation

**Figure 3:**
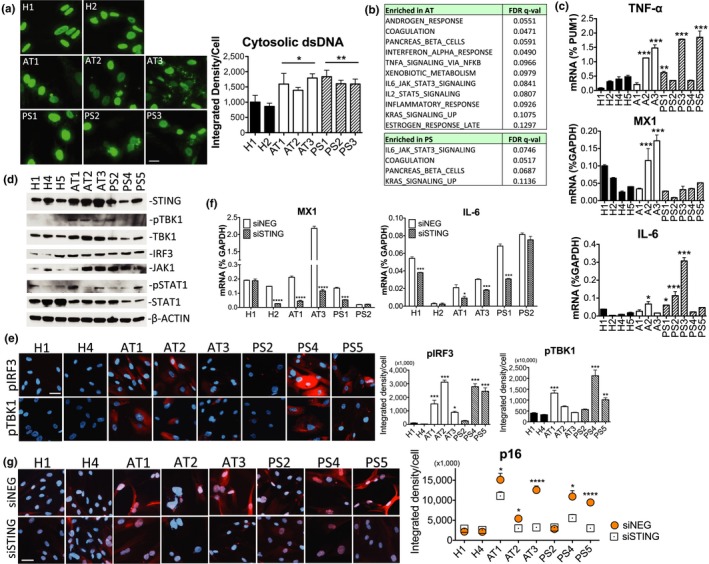
DNA accumulation and sensing in aging diseases. (a) IF staining and quantitation of anti‐dsDNA in healthy (H), ataxia (AT), and progeria (PS) skin fibroblasts. Numbers represent different fibroblasts of each genotype (Coriell catalog no. listed in Experimental Procedures). One‐way ANOVA among samples, **p = 0.0038, and significance of grouped genotype by *t* test as indicated. (b) Enriched hallmark gene sets in AT (top) and PS (bottom) by GSEA with FDR < 0.25. (c) TNF‐α, MX1 and IL‐6 transcript expression of H, AT, and PS fibroblasts assessed by RT‐qPCR, ****p < 0.0001 among samples by 1‐way ANOVA for all 3 genes. Asterisks indicate significance of individual cells versus H1 for TNF‐α, H4 and H5 for MX1, and all healthy cells for IL‐6 by Tukey's test. (d) Immunoblot showing DNA‐sensing mediators in H, AT, and PS cells, double bands in total STING are visible in some AT cells, β‐ACTIN, loading control. (e) IF staining and quantitation of pIRF3 and pTBK1 (both red signals) in H, AT, and PS cells, DAPI (blue), counterstain. Significance among samples for both phospho‐proteins, ****p < 0.0001, 1‐way ANOVA; and individual cells versus H1 and H4 by Tukey's test as indicated. (f–g) H, AT, and PS cells with *STING* knocked down by transfected siRNAs and assessed for MX1 and IL‐6 expressions by RT‐qPCR (f), and p16 expression (red) by IF staining (g). Two‐way ANOVA by genotype and *siSTING *in (g), ****p < 0.0001; DAPI (blue), counterstain. Asterisks denote significance of *siSTING *versus* siNEG* control in individual cells by *t* test. Results in a, c, d, e, f, and g are representative of 2 independent experiments

Consistent with our original model of extranuclear DNA being processed by autophagy and stimulating the STING pathway, we found that olds cells treated with bafilomycin A1 (which blocks lysosomal fusion to autophagosomes) showed increased levels of MX1 and CXCL10, while cells treated with rapamycin (that stimulates autophagy) reduced MX1 expression (Figure 2f). This is consistent with our finding that LC3 and LAMP1 are associated with exported DNA in aged cells (Figure S1E and G). Furthermore, when we knocked down genes in the cGAS‐STING‐TBK1 axis by siRNAs (Figure S2H, knockdown efficiency), expression of IFNI‐inducible MX1 and IFIT1 (Figure 2g) and 3 of 10 detectable SASP factors (Figure S2I) was reduced. Overall, these results indicate a heightened innate immune response in old cells, which is cGAS/STING‐dependent and is affected by autophagy and lysosomal activity.

### 2.5 Cytosolic DNA accumulates and inflammatory pathways are activated in cells from premature aging syndromes

We wondered if our hypothesis that cytosolic DNA is a cell‐intrinsic inflammatory ligand might extend to cells from humans with aging diseases. We focused on two genetic disorders, ataxia telangiectasia (AT), a severe neurodegenerative syndrome caused by gene defects in *ATM* (ataxia telangiectasia mutated) which is essential for DSB repair, and Hutchinson–Gilford progeria (HGPS or PS), which exhibits premature aging symptoms due to a mutation in *LAMIN A* (*LMNA*) that maintains nuclear architecture. Mutations of either disease gene, we reasoned, could lead to excess cytosolic DNA as a result of increased DNA damage (in AT) or leaky nuclear envelope (in PS). As hypothesized, we found prominent extranuclear dsDNA accumulation in the form of nuclear buds, speckles, and large fragments by anti‐dsDNA IF staining in different AT and PS skin fibroblasts of patients but not healthy donors (Figure 3a). DNA accumulation in both conditions also correlated with increased DSBs marked by γ‐H2AX (Figure S3A). We then examined the innate immune profiles of the healthy and disease fibroblasts (H1, 4, 5; AT1–3; PS1–5) by RNA‐seq. GSEA showed AT having 5 of 11 enriched gene sets in immune processes, including IFN‐α, TNF‐α, and IL‐6 responses, while PS showed increased IL‐6 signaling as the top enriched gene set (Figure 3b). By RT‐qPCR, we observed higher expression of TNF‐α, an important cytokine in inflammaging (Fagiolo et al., 1993; Roubenoff et al., 2003), in cells from both diseases and confirmed that AT and PS cells differentially upregulated genes downstream of the two innate immune arms (MX1 for the TBK1‐IRF3 arm, and IL‐6 for IKK‐NF‐κB) (Figure 3c, ****p < 0.0001, 1‐way ANOVA), consistent with GSEA. The increased innate immune responses are suggestive of activation of DNA sensing as a result of accumulated cytosolic DNA.

### 2.6 STING‐mediated induction of inflammation and p16 in AT and PS cells

Indeed, we observed in AT cells dramatic elevation of total STING abundance and concomitant increase in its downstream effectors pTBK1, TBK1, IRF3, and JAK1 (and pSTAT1 in some cells) by immunoblotting (Figure 3d). An increase in STING was also found in some PS cells (Figure S3B, additional PS cells). Phospho‐IRF3 was not detected by immunoblotting, but could be visualized by IF staining. Nuclear pIRF3 staining upon DNA stimulation was consistent with known pIRF3 responses (Figure S3C). In disease cells (AT1, AT2, AT3, PS4, and PS5), we observed cytosolic and nuclear patterns of pIRF3 distribution (Figure 3e). A previous study reported similar pIRF3‐containing signaling complexes accumulating in enlarged juxtanuclear recycling endosomes (Gorbea, Rechsteiner, Vallejo, & Bowles, 2013). Elevated levels of pIRF3 in disease cells were associated with an increase in pTBK1 IF signals (Figure 3e, ****p < 0.0001 for both pIRF3 and pTBK1, 1‐way ANOVA). When we knocked down *STING* by transfected siRNAs (Figure S3D, knockdown efficiency), expression of MX1 and IL‐6 was abrogated in AT cells (which also had higher STING protein levels), but the effect in PS cells was more variable (Figure 3f). To further test if DNA sensing is causal in the senescent phenotype of disease cells, we knocked down *STING* and found significant reduction of the senescence marker p16 in all AT and PS cells tested, except PS2 (Figure 3g). Basal levels of p16 in AT1, AT2, AT3, PS4, and PS5 were all significantly higher than those in healthy controls (***p < 0.005, Tukey's test). These results support a role for the STING pathway in inducing genes involved in inflammation and senescence in aging diseases with accumulated DNA.

### 2.7 DNA burden impacts age‐related inflammation

**Figure 4:**
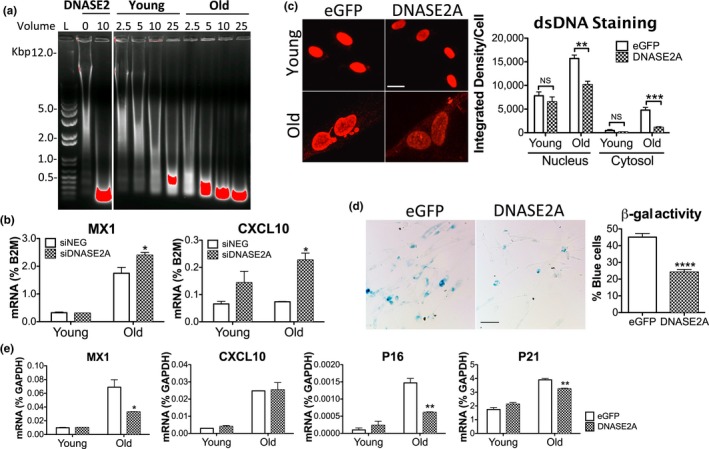
DNA burden impacts age‐related inflammation. (a) Digestion of 50 μg calf thymus DNA by cell lysates (μl) from 2 million young or old MRC5 cells. Degraded DNA fragments visualized on 0.7% agarose gel by ethidium bromide. Recombinant DNASE2 (10 μg/ml) used as positive control. L, DNA ladder; red denotes saturated amounts of DNA. (b) MX1 and CXCL10 mRNA expression assessed by RT‐qPCR in young and old MRC5 cells after knocking down *DNASE2A* using transfected siRNAs; *t* test significance relative to *siNEG*, nontargeting control. (c–e) Young and old MRC5 cells transduced with DNASE2A ORF for constitutive overexpression, and examined for anti‐dsDNA staining by IF (c), SA‐β‐gal activity (d), and expression of inflammatory and cell‐cycle genes by RT‐qPCR (e). EGFP, negative control; scale bar, 20 μm in (c) and 50 μm in (d). Significance based on eGFP values by *t* test. All data are representative of at least 2 independent experiments

On a per cell basis, lysates from old cells showed a stronger capacity to degrade dsDNA than young cells, reflecting an elevated clearance of excess DNA by lysosomal DNASE2A (Figure 4a). We reasoned that increased DNA degradation might restore the younger cell phenotype or even revert the process of senescence. As expected, knocking down *DNASE2A* in MRC5 cells worsened inflammation in old cells, resulting in increased MX1 and CXCL10 (Figure 4b), and heightened SASP factors such as GM‐CSF, IGFBP7, MMP3 (Figure S4A). We then overexpressed the DNA degrading enzyme in cells by a constitutive lentiviral vector encoding the *DNASE2A* open reading frame (ORF). *DNASE2A* transduced in old MRC5 cells (Figure S4B, expression by RT‐qPCR) significantly reduced cytosolic and even nuclear DNA (Figure 4c), SA‐β‐gal activity (Figure 4d), and expression of MX1 and genes controlling cell cycle (p16, p21) (Figure 4e). Unfortunately, we were unable to revive cell growth in old cells by increasing DNA degradation alone. Indeed, old cells appeared to arrest their growth prematurely in response to viral transduction, suggesting that *DNASE2A *will need to be delivered using a different method. Also, converting cellular senescence to an active proliferative state may prove difficult since other factors may make it irreversible. We conclude that excess DNA strongly impacts the senescence response in vitro, and that its removal can alleviate inflammation in old cells.

### 2.8 Dnase2a deficiency recapitulates cellular senescence

**Figure 5:**
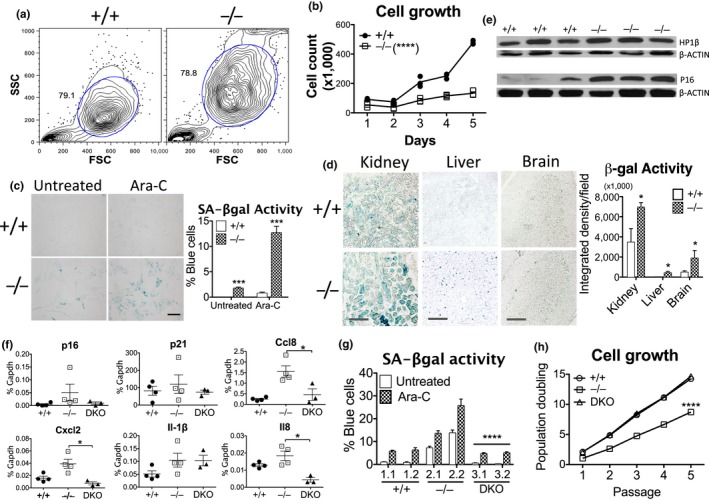
*Dnase2a* deficiency recapitulates cellular senescence. (a) Contour plots showing FSC (forward scatter) for cell size and SSC (side scatter) for granularity in *Dnase2a* ^+/+^ and *Dnase2a^−^* ^/^ *^−^* MLFs. (b) Cell proliferation of *Dnase2a* ^+/+^ and *Dnase2a^−^* ^/^ *^−^* MLFs by manual count with trypan blue, ****p < 0.0001 by phenotype, 2‐way ANOVA. (c) SA‐β‐gal activity and quantitation of *Dnase2a* ^+/+^ and *Dnase2a^−^* ^/^ *^−^* MLFs without or with Ara‐C treatment (10 μM, 24 hr); scale bar, 50 μm. (d) Representative SA‐β‐gal staining of *Dnase2a* ^+/+^ and *Dnase2a^−^* ^/^ *^−^* mouse tissues as indicated. Quantitation based on 5 random fields of 5× or 10× images of representative pair of mice, scale bar, 100 μm in kidney and liver, 200 μm in brain. (e) Immunoblot of HP1β and p16 in *Dnase2a* ^+/+^ and *Dnase2a^−^* ^/^ *^−^* kidney tissues, β‐ACTIN, loading control. (f) Transcript expression of cell‐cycle genes and SASP factors in kidney tissues of *Dnase2a* ^+/+^ (*n* = 4), *Dnase2a^−^* ^/^ *^−^* (*n* = 4), and *Dnase2a;Sting* double KO (DKO) (*n* = 3) mice by RT‐qPCR. (g) Quantitation of SA‐β‐gal activity in *Dnase2a* ^+/+^, *Dnase2a^−^* ^/^ *^−^*, and DKO MLFs, untreated or treated with Ara‐C (10 μM, 24 hr). Numbers indicate single clones of each genotype. ****p < 0.0001, for genotype and Ara‐C‐treatment by 2‐way ANOVA, and *t* test between *Dnase2a^−^* ^/^ *^−^* and DKO MLFs as indicated. (h) Cell growth over serial passage in *Dnase2a* ^+/+^, *Dnase2a^−^* ^/^ *^−^*, and DKO MLFs where equal number of cells are re‐plated at each split. Effect by genotype between *Dnase2a^−^* ^/^ *^−^* and DKO, ****p < 0.0001, 2‐way ANOVA. Data are representative of 2 independent experiments in a–c, g, h, and 3 pairs of age and sex‐matched littermates in d, e. *p*‐value of significance by *t* test or as indicated

While our results are consistent with a model for DNA‐induced inflammation and senescence in old cells and premature aging disease cells, we did not conclusively show that DNA levels or STING‐mediated sensing of intrinsic DNA affect senescence. We thus turned back to the genetic mouse model in which we previously studied the transport of excess nuclear DNA to the lysosomes for degradation by DNASE2A. To test whether DNA accumulation resulting from *Dnase2a* deficiency can promote a senescent phenotype, we examined cellular features of senescence including cell morphology, growth rates and response to stimuli. Compared with *Dnase2a* ^+/+^ cells, *Dnase2a^−^* ^/^ *^−^* mouse lung fibroblasts (MLFs) showed increased size and granularity by flow cytometry (Figure 5a), and slower growth by cell count (Figure 5b) or the proliferation marker Ki67 (Figure S5A). SASP factors were upregulated in *Dnase2a^−^* ^/^ *^−^* MLFs (Figure S5B) and tissues, including kidney and heart (Figure S5C), compared to *Dnase2a* ^+/+^ mice as assessed by RT‐qPCR. *Dnase2a^−^* ^/^ *^−^* cells also showed a higher percentage of cells with SA‐βgal activity in baseline or in response to Ara‐C treatment, compared to *Dnase2a* ^+/+^ cells (Figure 5c)—resembling the phenotype seen in response to DNA damage in old human cells (Figure S1F). In various tissues, such as kidney, liver, and brain, SA‐β‐gal activity was stronger in *Dnase2a^−^* ^/^ *^−^* than *Dnase2a* ^+/+^ mice (Figure 5d). Increased SA‐β‐gal activity also correlated with higher protein expression of the aging markers heterochromatin protein 1β (HP1β) and p16 in the kidney of *Dnase2a^−^* ^/^ *^−^* mice (Figure 5e), though concomitant increase in p53 failed to elevate p21 expression (Figure S5D), suggesting additional regulation of this downstream effector. Higher p16 levels were similarly observed in less actively renewed tissues of brain and heart in *Dnase2a^−^* ^/^ *^−^* mice by immunohistochemical staining (Figure S5E). We examined the requirement for DNA sensing using *Dnase2a^−^* ^/^ *^−^*;*Sting^−^* ^/^ *^−^* double KO (DKO) mice and found reduced gene expression of p16 and SASP factors such as Ccl8, Cxcl12, and Il8 in kidney tissues (Figure 5f). DKO MLFs also showed lower SA‐β‐gal activity especially upon Ara‐C treatment, compared with *Dnase2a^−^* ^/^ *^−^* cells (Figure 5g), and importantly, regained proliferation like *Dnase2a* ^+/+^ cells (Figure 5h). The rescued phenotype in DKO thus supports the central role of STING in promoting senescence upon excess cytosolic DNA accumulation.

## 3 DISCUSSION

In this study, we address the hypothesis that sensing and degradation of self‐DNA impact inflammation in cells undergoing replicative senescence or cells derived from aging diseases. First, in late passage cells, we observed DNA exported from the nucleus through a leptomycin B‐sensitive pathway and showed that inflammatory cytokines were induced through the STING pathway. Second, we found that inflammation could be reduced by overexpression of DNASE2A. Finally, using a mouse model, we found that excess DNA burden led to STING‐dependent inflammation and senescence phenotype with reduced proliferation in mouse cells ex vivo and mouse tissues in vivo*.* Our data support a model in which extranuclear DNA triggers inflammation and senescence in vivo and in several cellular models of aging.

In our previous studies of autoimmune *Dnase2a* knockout mice, we found that cell‐intrinsic activation of innate immune responses via the STING pathway occurred when excessive damaged nuclear DNA overwhelmed lysosomal degradation of DNA transported by autophagy (Lan et al., 2014). We proposed that intrinsic damaged DNA accumulation could also trigger inflammation during aging, cancer, and chemotherapy. Consistent with this model, we and Dou et al. (Dou et al., 2017) both observed cytoplasmic chromatin fragments in the form of nuclear buds and aggregates in old cells—though we also found smaller DNA speckles by dsDNA staining. Furthermore, several studies have recently shown that excess cytosolic DNA activates the paracrine SASP program and cellular senescence via cGAS‐STING (Dou et al., 2017; Glück et al., 2017; Yang et al., 2017). We confirmed some of the findings of these groups in human cells undergoing replicative senescence and further validated the model in a mouse that lacks *Dnase2a *or *Dnase2a *and *Sting*, allowing us to show that excess cellular DNA in *Dnase2‐*deficient mice increases age‐associated inflammation and senescence in a *Sting*‐dependent fashion.

A critical question is what gives rise to the excess damaged DNA in older cells. DNA lesions can be caused by replication errors, radiation, oxygen, bacterial infection, and oncogenes (Bartkova et al., 2006; Di Leonardo, Linke, Clarkin, & Wahl, 1994; Nougayrede et al., 2006; Parrinello et al., 2003). Toxic DSBs are associated with senescence and inflammation in aged human tissues (Lu et al., 2004), and we observed high TUNEL or γ‐H2AX staining across late passage human fibroblasts, *Dnase2a *KO mouse cells, and AT or PS patient cells. In ATM‐deficient cells, cytosolic single‐stranded DNAs are found to activate the STING pathway (Hartlova et al., 2015). However, much more work is needed to understand the sources of excess DNA and damaged DNA in aging‐related conditions.

A second question is how DNA is exported from the nucleus and appears in the cytosol to be detected by DNA sensors. Weakened or altered nuclear envelope in old cells can facilitate such an escape. Deteriorated nuclear architecture is also observed in nuclear envelopathies and laminopathies—HGPS a typical example with aggregation or absence of nuclear lamina proteins or NPC (Goldman et al., 2004). For DNA export, we postulate three options: NPC export, nuclear egress, and nuclear rupture. First, our data with leptomycin blocking nuclear export show that at least in part this nuclear export pathway overlaps with the DNA export pathway. Second, an endogenous egress pathway, which overcomes the NPC size limit, used in nuclear budding by DNA viruses is now reported for nuclear export of large ribonucleoproteins (Speese et al., 2012). Third, in situations of defective or severely incomplete nuclear perimeter, transient nonlethal nuclear envelope ruptures can take place in several contexts, including cancer cells, laminopathies, migrating cells, and micronuclei (De Vos et al., 2011; Hatch, Fischer, Deerinck, & Hetzer, 2013; Raab et al., 2016; Vargas, Hatch, Anderson, & Hetzer, 2012), which could allow simple diffusion of DNA to the cytosol.

A third problem is what form of DNA is sensed by what mechanisms. The origin (hot spots, fragile sites, microsatellites), sequence, length, and form (naked, protein‐bound) of the extruded DNA are still unknown. It is generally accepted that immunostimulatory DNA is sequence‐independent, though a cGAS recognition motif has been identified (Herzner et al., 2015). We also do not know if activation of the pathway is proportional to the quantity of DNA accumulated—basal versus induced levels of cytokines in old cells are 2‐ to 4‐fold vs 4‐ to 10‐fold in AT and PS cells. Interactions of DNA sensing with other processes, such as autophagic and lysosomal degradation, are also possible, for example cGAS can interact with Beclin‐1 to halt excessive immune activation (Liang et al., 2014). Furthermore, DNA sensors and regulators are not static within the cell. cGAS localizes to the site of damaged DNA, for example nucleus during mitosis (Yang et al., 2017) and cytosol after damage (Glück et al., 2017). STING remains largely cytosolic upon DNA stimulation, but is also found in the ER, Golgi, nuclear membrane, autophagic vesicles, and exosomes. Another DNA sensor IFI16, also upregulated in old cells and AT cells (Duan et al., 2011), translocates between nucleus and cytosol and interacts with cGAS (Almine et al., 2017) or the AIM2 inflammasome (Kerur et al., 2011). We expect many more layers of sensing and regulation to link DNA damage with senescence and aging.

A final gap in our knowledge is how DNA‐induced inflammation contributes to the phenotypes of aging. IFNI signaling is important in oncogene‐induced senescence (Katlinskaya et al., 2016), aging model of *Terc *mice (Yu et al., 2015), and aged human and mouse brains (Baruch et al., 2014). However, our results in old cells reveal not only upregulated but also downregulated type I IFN‐regulated DEGs. The aging inflammatory signature may thus turn out to be more nuanced than simply IFN response or SASP. Nonetheless, the two inflammatory arms of the STING pathway are active in AT (IFNI) and PS (NF‐κB/IL‐6), respectively. In vivo, loss of STING can revert senescent defects in cells and tissues from Dnase2*^−^* ^/^ *^−^* mice, as well as rescue proliferation defects. More mechanistic studies will help explain how DNA‐mediated responses and processes are connected to senescence pathways in cells from later passage, or AT and PS patients and in vivo.

In vitro replicative senescence serves as a simplified model here. A limitation is that we observe heterogeneity in inflammatory signatures across three established cell lines, likely due to intrinsic (e.g., genetic) and extrinsic (passage number, culture conditions) variability. For example, whereas loss of cGAS is reported to reduce a few particular SASP factors (IL‐6, IL‐8, TNF‐α) in three recent studies (Dou et al., 2017; Glück et al., 2017; Yang et al., 2017), we saw variable responses of 10 detectable factors in old cells when knocking down STING (Figure S2I). This variability highlights the need to study many more healthy and disease primary cells before generalizing the findings or extrapolating to human organismal aging. Enhancing degradation of excess DNA by overexpressing DNASE2A or by triggering autophagy could reduce inflammation in aged cells and disease AT and PS cells, but old cells did not start to grow. Takahashi et al. were also unable to revive cell‐cycle arrest in Ras‐induced senescence by ectopic DNASE2 or TREX1 (Takahashi et al., 2018). These suggest technical hurdles in removing excess DNA sufficiently or the existence of other factors that induce or maintain senescence.

If self‐DNA contributes to aging‐associated inflammation and cellular senescence, then modulating any step in the process we elucidated (i.e., DNA damage, transport, sensing and degradation) may be therapeutically useful. Beyond conventional anti‐inflammatory drugs (such as aspirin and NSAIDs), we propose that reducing DNA in the cytosolic environment could reduce inflammation and act as a novel therapeutic strategy for treating degenerative and aging‐associated diseases, especially laminopathies with nuclear DNA found in the cytosol, and interferonopathies caused by loss of degradation components (Rodero et al., 2017).

## 4 EXPERIMENTAL PROCEDURES

### 4.1 Cell lines and culture

IMR90, MRC5, and WI38 cells were cultured in DMEM plus 15% FBS, 1% penicillin/streptomycin, l‐glutamate and sodium bicarbonate under 3% O_2_ and 6% CO_2_ at 37°C to limit oxidative stress from atmospheric oxygen. Cells were split at 80%–90% confluence and PD calculated as log_2_ (no. at split/no. plated). Young cells were <PD30 and old cells were from splits >10 days in culture (often last 3 splits before permanent growth arrest), for example PD67–70 in MRC5 cells. Human diploid fibroblast cultures derived from donor, AT and PS patient skins were obtained from Coriell Institute (Camden, NJ, USA), and maintained in DMEM, 15% FBS, 1% penicillin/streptomycin and l‐glutamate under 5% CO_2_ at 37°C. Healthy donor cells are H1: AG04392, PD17–30; H2: AG04433, PD12–28; H4: AG04525, PD13–25; H5: AG06555, PD19–35; AT cells are AT1: AG02496, PD unknown; AT2: AG03058, PD12–19; AT3: AG04405, PD9–18; and PS cells are PS1: AG10578, PD6–13; PS2: AG11513, PD9–16; PS3: AG03513, PD15–21; PS4: AG00989, PD27–34; PS5: AG019732, PD28–35. Splits of actively dividing passages were used, when H cells took 4–7 days, AT and PS cells 8–14 days.

### 4.2 Immunofluorescence cell staining and quantitation

Cells were cultured in Lab‐Tek II 8‐well chamber slides, fixed with 4% PFA (10 min), permeabilized with 0.5% Triton X‐100 (5 min), then blocked and stained with antibodies against: dsDNA (Santa Cruz, 1:500), γH2AX, NUP98, pTBK1 (both Cell Signaling, 1:200), LC3 (Novus Biologicals, 1:200) or biotin‐LAMP1 (BioLegend, 1:200), followed by fluorescent secondary antibodies (45 min). Methanol fixation was used for anti‐pIRF3 (Cell Signaling, 1:200) staining.

Nuclear and cytosolic DNA signals from 5 of 10× or 20× images (~50–200 cells per image) were quantified using Fiji. Region of interest (ROI) was defined in each image and threshold set to measure fluorescence above background. Nuclear ROI was defined by DAPI staining and its intensity subtracted from total fluorescence to determine cytoplasmic signal. Quantitative fluorescence was presented as integrated density per cell with cell number determined by particle count of DAPI.

### 4.3 *DNASE2A* overexpression

Human *DNASE2A* ORF or control eGFP was cloned into a constitutive pLX304 vector with blasticidin resistance (Genetic Perturbation Platform, Broad Institute). Plasmid DNA was purified and transfected into 293 cells for packaging of lentiviruses, and viruses produced were used to infect human fibroblasts. Blasticidin selection was at 10 μg/ml, 24–48 hr.

Additional details for Experimental Procedures are supplied in Supporting Information Appendix S1.

## CONFLICT OF INTEREST

The authors declare no conflict of interest.

## AUTHOR CONTRIBUTIONS

Y.Y.L. and N.H. conceived the project, designed experimental strategies, and revised the manuscript for publication. Y.Y.L. prepared the first draft of the manuscript and performed experiments and analysis to completion. J.M.H. analyzed expression data from RNA‐seq and NanoString and helped editing. T.E. processed RNA‐seq samples. C.S.G. performed initial experiments during his two‐month Harvard Medical School graduate student rotation in the Hacohen lab. D.L. aligned expression data and assisted analysis and discussions. R.R performed immunoblots for paper revision.

## References

[acel12901-bib-0001] Almine, J. F. , O'Hare, C. A. , Dunphy, G. , Haga, I. R. , Naik, R. J. , Atrih, A. , … Unterholzner, L. (2017). IFI16 and cGAS cooperate in the activation of STING during DNA sensing in human keratinocytes. Nature Communications, 8, 14392 10.1038/ncomms14392. PMC5316833 28194029

[acel12901-bib-0002] Bartkova, J. , Rezaei, N. , Liontos, M. , Karakaidos, P. , Kletsas, D. , Issaeva, N. , … Gorgoulis, V. G. (2006). Oncogene‐induced senescence is part of the tumorigenesis barrier imposed by DNA damage checkpoints. Nature, 444(7119), 633–637. 10.1038/nature05268. 17136093

[acel12901-bib-0003] Baruch, K. , Deczkowska, A. , David, E. , Castellano, J. M. , Miller, O. , Kertser, A. , … Schwartz, M. (2014). Aging‐induced type I interferon response at the choroid plexus negatively affects brain function. Science, 346(6205), 89–93. 10.1126/science.1252945. 25147279 PMC4869326

[acel12901-bib-0004] Coppé, J. P. , Patil, C. K. , Rodier, F. , Krtolica, A. , Beausejour, C. M. , Parrinello, S. , … Campisi, J. (2010). A human‐like senescence‐associated secretory phenotype is conserved in mouse cells dependent on physiological oxygen. PLoS One, 5(2), e9188 10.1371/journal.pone.0009188. 20169192 PMC2820538

[acel12901-bib-0005] De Vos, W. H. , Houben, F. , Kamps, M. , Malhas, A. , Verheyen, F. , Cox, J. , … Broers, J. L. (2011). Repetitive disruptions of the nuclear envelope invoke temporary loss of cellular compartmentalization in laminopathies. Human Molecular Genetics, 20(21), 4175–4186. 10.1093/hmg/ddr344. 21831885

[acel12901-bib-0006] Di Leonardo, A. , Linke, S. P. , Clarkin, K. , & Wahl, G. M. (1994). DNA damage triggers a prolonged p53‐dependent G1 arrest and long‐term induction of Cip1 in normal human fibroblasts. Genes & Development, 8(21), 2540–2551. 10.1101/gad.8.21.2540 7958916

[acel12901-bib-0007] Dou, Z. , Ghosh, K. , Vizioli, M. G. , Zhu, J. , Sen, P. , Wangensteen, K. J. , … Berger, S. L. (2017). Cytoplasmic chromatin triggers inflammation in senescence and cancer. Nature, 550(7676), 402–406. 10.1038/nature24050. 28976970 PMC5850938

[acel12901-bib-0008] Duan, X. , Ponomareva, L. , Veeranki, S. , Panchanathan, R. , Dickerson, E. , & Choubey, D. (2011). Differential roles for the interferon‐inducible IFI16 and AIM2 innate immune sensors for cytosolic DNA in cellular senescence of human fibroblasts. Molecular Cancer Research, 9(5), 589–602. 10.1158/1541-7786.MCR-10-0565. 21471287 PMC3096691

[acel12901-bib-0009] Fagiolo, U. , Cossarizza, A. , Scala, E. , Fanales‐Belasio, E. , Ortolani, C. , Cozzi, E. , … Paganelli, R. (1993). Increased cytokine production in mononuclear cells of healthy elderly people. European Journal of Immunology, 23(9), 2375–2378. 10.1002/eji.1830230950. 8370415

[acel12901-bib-0010] Glück, S. , Guey, B. , Gulen, M. F. , Wolter, K. , Kang, T. W. , Schmacke, N. A. , … Ablasser, A. (2017). Innate immune sensing of cytosolic chromatin fragments through cGAS promotes senescence. Nature Cell Biology, 19(9), 1061–1070. 10.1038/ncb3586. 28759028 PMC5826565

[acel12901-bib-0011] Goldman, R. D. , Shumaker, D. K. , Erdos, M. R. , Eriksson, M. , Goldman, A. E. , Gordon, L. B. , … Collins, F. S. (2004). Accumulation of mutant lamin A causes progressive changes in nuclear architecture in Hutchinson‐Gilford progeria syndrome. Proceedings of the National Academy of Sciences, 101(24), 8963–8968. 10.1073/pnas.0402943101. PMC428455 15184648

[acel12901-bib-0012] Gorbea, C. , Rechsteiner, M. , Vallejo, J. G. , & Bowles, N. E. (2013). Depletion of the 26S proteasome adaptor Ecm29 increases Toll‐like receptor 3 signaling. Science Signalling, 6(295), ra86 10.1126/scisignal.2004301. 24084648

[acel12901-bib-0013] Hartlova, A. , Erttmann, S. F. , Raffi, F. A. , Schmalz, A. M. , Resch, U. , Anugula, S. , … Gekara, N. O. (2015). DNA damage primes the type I interferon system via the cytosolic DNA sensor STING to promote anti‐microbial innate immunity. Immunity, 42(2), 332–343. 10.1016/j.immuni.2015.01.012. 25692705

[acel12901-bib-0014] Hatch, E. M. , Fischer, A. H. , Deerinck, T. J. , & Hetzer, M. W. (2013). Catastrophic nuclear envelope collapse in cancer cell micronuclei. Cell, 154(1), 47–60. 10.1016/j.cell.2013.06.007. 23827674 PMC3749778

[acel12901-bib-0015] Herzner, A. M. , Hagmann, C. A. , Goldeck, M. , Wolter, S. , Kubler, K. , Wittmann, S. , … Schlee, M. (2015). Sequence‐specific activation of the DNA sensor cGAS by Y‐form DNA structures as found in primary HIV‐1 cDNA. Nature Immunology, 16(10), 1025–1033. 10.1038/ni.3267. 26343537 PMC4669199

[acel12901-bib-0016] Ivanov, A. , Pawlikowski, J. , Manoharan, I. , van Tuyn, J. , Nelson, D. M. , Rai, T. S. , … Adams, P. D. (2013). Lysosome‐mediated processing of chromatin in senescence. Journal of Cell Biology, 202(1), 129–143. 10.1083/jcb.201212110. 23816621 PMC3704985

[acel12901-bib-0017] Katlinskaya, Y. V. , Katlinski, K. V. , Yu, Q. , Ortiz, A. , Beiting, D. P. , Brice, A. , … Fuchs, S. Y. (2016). Suppression of Type I Interferon Signaling Overcomes Oncogene‐Induced Senescence and Mediates Melanoma Development and Progression. Cell Reports, 15(1), 171–180. 10.1016/j.celrep.2016.03.006. 27052162 PMC4826807

[acel12901-bib-0018] Kerur, N. , Veettil, M. V. , Sharma‐Walia, N. , Bottero, V. , Sadagopan, S. , Otageri, P. , & Chandran, B. (2011). IFI16 acts as a nuclear pathogen sensor to induce the inflammasome in response to Kaposi Sarcoma‐associated herpesvirus infection. Cell Host & Microbe, 9(5), 363–375. 10.1016/j.chom.2011.04.008. 21575908 PMC3113467

[acel12901-bib-0019] Lan, Y. Y. , Londono, D. , Bouley, R. , Rooney, M. S. , & Hacohen, N. (2014). Dnase2a deficiency uncovers lysosomal clearance of damaged nuclear DNA via autophagy. Cell Reports, 9(1), 180–192. 10.1016/j.celrep.2014.08.074. 25284779 PMC4555847

[acel12901-bib-0020] Le, O. N. , Rodier, F. , Fontaine, F. , Coppé, J. P. , Campisi, J. , DeGregori, J. , … Beausejour, C. M. (2010). Ionizing radiation‐induced long‐term expression of senescence markers in mice is independent of p53 and immune status. Aging Cell, 9(3), 398–409. 10.1111/j.1474-9726.2010.00567.x. 20331441 PMC2894262

[acel12901-bib-0021] Li, T. , & Chen, Z. J. (2018). The cGAS‐cGAMP‐STING pathway connects DNA damage to inflammation, senescence, and cancer. Journal of Experimental Medicine, 215(5), 1287–1299. 10.1084/jem.20180139. 29622565 PMC5940270

[acel12901-bib-0022] Liang, Q. , Seo, G. J. , Choi, Y. J. , Kwak, M. J. , Ge, J. , Rodgers, M. A. , … Jung, J. U. (2014). Crosstalk between the cGAS DNA sensor and Beclin‐1 autophagy protein shapes innate antimicrobial immune responses. Cell Host & Microbe, 15(2), 228–238. 10.1016/j.chom.2014.01.009. 24528868 PMC3950946

[acel12901-bib-0023] Lu, T. , Pan, Y. , Kao, S. Y. , Li, C. , Kohane, I. , Chan, J. , & Yankner, B. A. (2004). Gene regulation and DNA damage in the ageing human brain. Nature, 429(6994), 883–891. 10.1038/nature02661. 15190254

[acel12901-bib-0024] Nougayrede, J. P. , Homburg, S. , Taieb, F. , Boury, M. , Brzuszkiewicz, E. , Gottschalk, G. , … Oswald, E. (2006). Escherichia coli induces DNA double‐strand breaks in eukaryotic cells. Science, 313(5788), 848–851. 10.1126/science.1127059. 16902142

[acel12901-bib-0025] Parrinello, S. , Samper, E. , Krtolica, A. , Goldstein, J. , Melov, S. , & Campisi, J. (2003). Oxygen sensitivity severely limits the replicative lifespan of murine fibroblasts. Nature Cell Biology, 5(8), 741–747. 10.1038/ncb1024. 12855956 PMC4940195

[acel12901-bib-0026] Raab, M. , Gentili, M. , de Belly, H. , Thiam, H. R. , Vargas, P. , Jimenez, A. J. , … Piel, M. (2016). ESCRT III repairs nuclear envelope ruptures during cell migration to limit DNA damage and cell death. Science, 352(6283), 359–362. 10.1126/science.aad7611. 27013426

[acel12901-bib-0027] Rodero, M. P. , Tesser, A. , Bartok, E. , Rice, G. I. , Della Mina, E. , Depp, M. , … Crow, Y. J. (2017). Type I interferon‐mediated autoinflammation due to DNase II deficiency. Nature Communications, 8(1), 2176 10.1038/s41467-017-01932-3. PMC5736616 29259162

[acel12901-bib-0028] Roubenoff, R. , Parise, H. , Payette, H. A. , Abad, L. W. , D'Agostino, R. , Jacques, P. F. , … Harris, T. B. (2003). Cytokines, insulin‐like growth factor 1, sarcopenia, and mortality in very old community‐dwelling men and women: The Framingham Heart Study. American Journal of Medicine, 115(6), 429–435. 10.1016/j.amjmed.2003.05.001 14563498

[acel12901-bib-0029] Rube, C. E. , Fricke, A. , Widmann, T. A. , Furst, T. , Madry, H. , Pfreundschuh, M. , & Rube, C. (2011). Accumulation of DNA damage in hematopoietic stem and progenitor cells during human aging. PLoS One, 6(3), e17487 10.1371/journal.pone.0017487. 21408175 PMC3049780

[acel12901-bib-0030] Rusinova, I. , Forster, S. , Yu, S. , Kannan, A. , Masse, M. , Cumming, H. , … Hertzog, P. J. (2013). Interferome v2. 0: An updated database of annotated interferon‐regulated genes. Nucleic Acids Research, 41(D1), D1040–D1046. 10.1093/nar/gks1215. 23203888 PMC3531205

[acel12901-bib-0031] Sedelnikova, O. A. , Horikawa, I. , Zimonjic, D. B. , Popescu, N. C. , Bonner, W. M. , & Barrett, J. C. (2004). Senescing human cells and ageing mice accumulate DNA lesions with unrepairable double‐strand breaks. Nature Cell Biology, 6(2), 168–170. 10.1038/ncb1095. 14755273

[acel12901-bib-0032] Singh, T. , & Newman, A. B. (2011). Inflammatory markers in population studies of aging. Ageing Research Reviews, 10(3), 319–329. 10.1016/j.arr.2010.11.002. 21145432 PMC3098911

[acel12901-bib-0033] Speese, S. D. , Ashley, J. , Jokhi, V. , Nunnari, J. , Barria, R. , Li, Y. , … Budnik, V. (2012). Nuclear envelope budding enables large ribonucleoprotein particle export during synaptic Wnt signaling. Cell, 149(4), 832–846. 10.1016/j.cell.2012.03.032. 22579286 PMC3371233

[acel12901-bib-0034] Takahashi, A. , Loo, T. M. , Okada, R. , Kamachi, F. , Watanabe, Y. , Wakita, M. , … Hara, E. (2018). Downregulation of cytoplasmic DNases is implicated in cytoplasmic DNA accumulation and SASP in senescent cells. Nature Communications, 9(1), 1249 10.1038/s41467-018-03555-8. PMC5871854 29593264

[acel12901-bib-0035] Vargas, J. D. , Hatch, E. M. , Anderson, D. J. , & Hetzer, M. W. (2012). Transient nuclear envelope rupturing during interphase in human cancer cells. Nucleus, 3(1), 88–100. 10.4161/nucl.18954. 22567193 PMC3342953

[acel12901-bib-0036] Wang, C. , Jurk, D. , Maddick, M. , Nelson, G. , Martin‐Ruiz, C. , & von Zglinicki, T. (2009). DNA damage response and cellular senescence in tissues of aging mice. Aging Cell, 8(3), 311–323. 10.1111/j.1474-9726.2009.00481.x. 19627270

[acel12901-bib-0037] Yang, H. , Wang, H. , Ren, J. , Chen, Q. , & Chen, Z. J. (2017). cGAS is essential for cellular senescence. Proceedings of the National Academy of Sciences, 114(23), E4612–E4620. 10.1073/pnas.1705499114. PMC5468617 28533362

[acel12901-bib-0038] Yu, Q. , Katlinskaya, Y. V. , Carbone, C. J. , Zhao, B. , Katlinski, K. V. , Zheng, H. , … Fuchs, S. Y. (2015). DNA‐damage‐induced type I interferon promotes senescence and inhibits stem cell function. Cell Reports, 11(5), 785–797. 10.1016/j.celrep.2015.03.069. 25921537 PMC4426031

