# Supplementary material for: Extranuclear DNA accumulates in aged cells and contributes to senescence and inflammation
Source: Aging Cell. 2019 Jan 31;18(2):e12901. doi: 10.1111/acel.12901 (PMC6413746; doi:10.1111/acel.12901)
Supplement: Supplementary file 1 [file ACEL-18-e12901-s001.docx]

# A B C


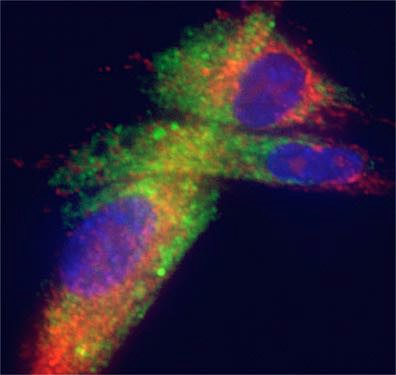

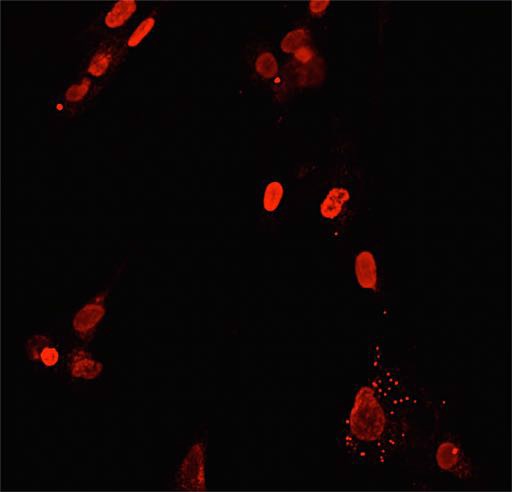

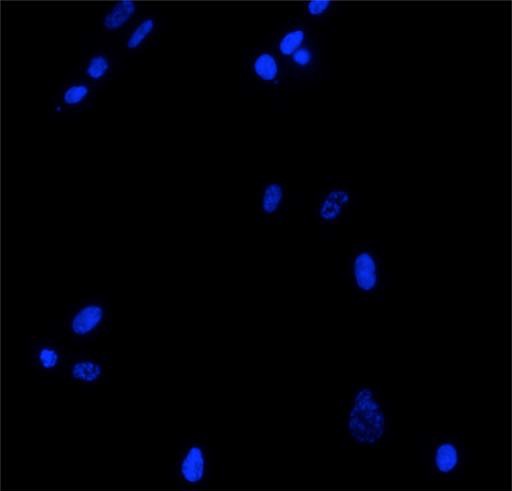

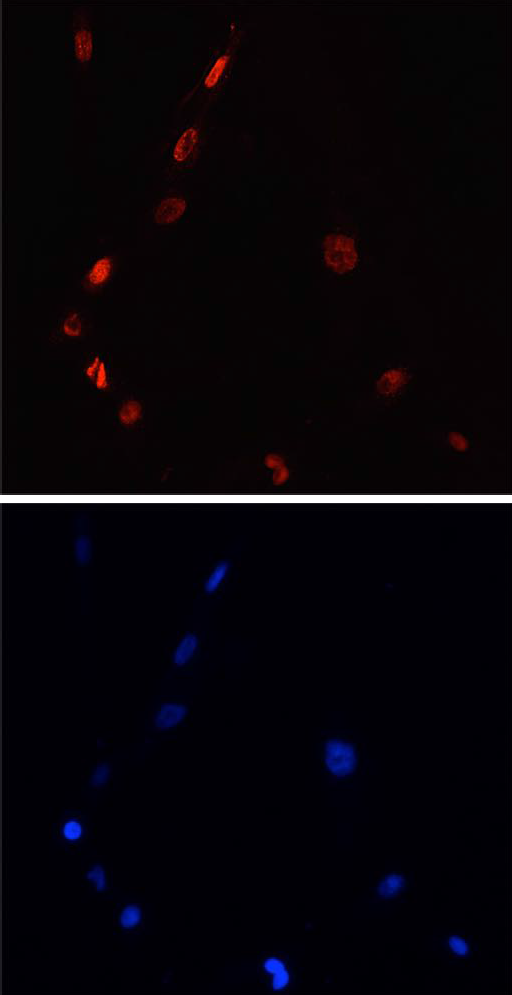


Untreated DNase1

dsDNA

**mRNA (%** β**-actin)**

150

100

**Autophagy Genes**

***


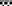

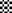

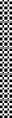

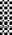

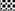


Young Old

50 **

* ***

DAPI

0

ATG5 BECN1 P62 PTEN

**D** 30,000


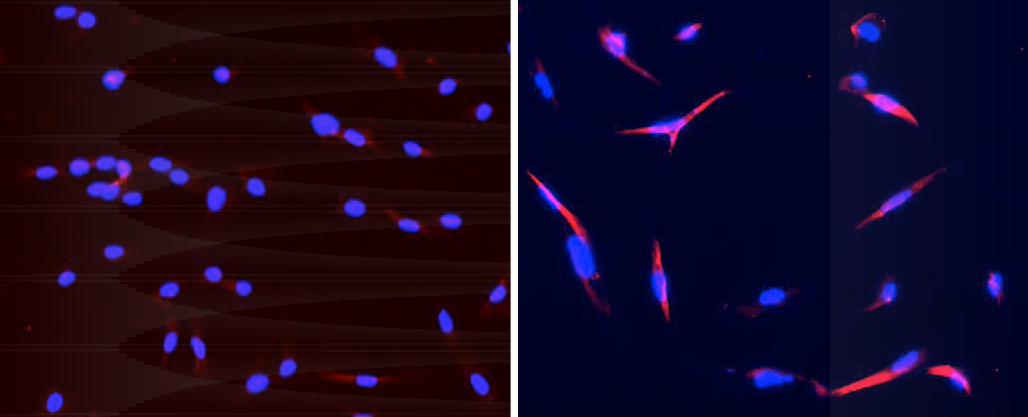

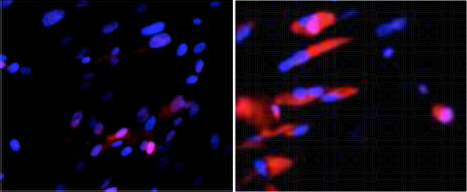


Young

Old

**Integrated Density/Cell**

**LC3_IF**


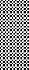

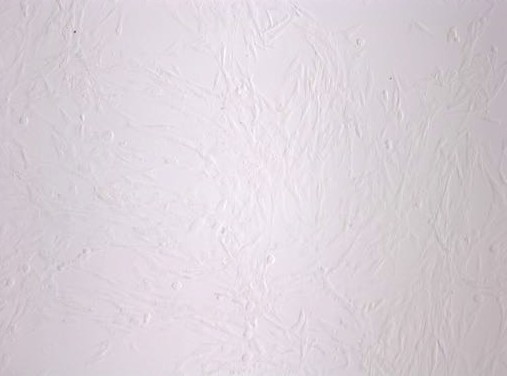

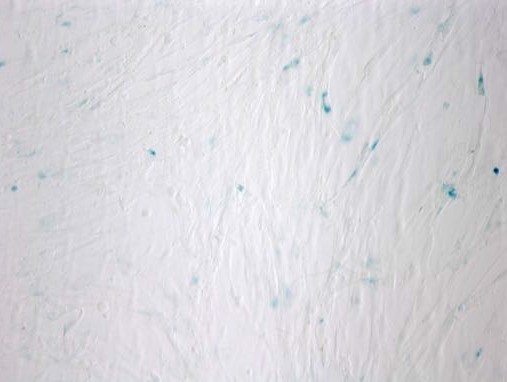

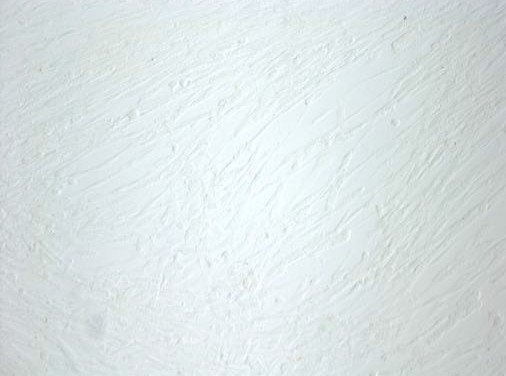

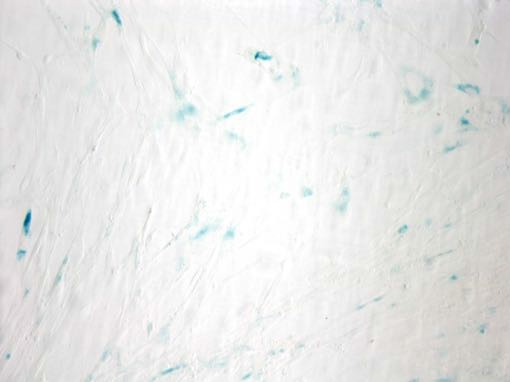


Untreated

AraGC

*** **F**

20,000

10,000

LC3

0

30,000

LAMP1

**Integrated Density/Cell**

20,000

10,000

Young Old

**LAMP1_IF**

***


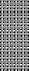


**SA-βgal Activity**

15 Ara-C 10


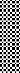

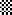


Young

**% Blue cells**

5

Old

0

Young Old

0

Young Old

1. dsDNA DAPI Merge


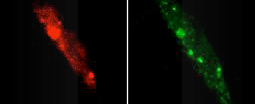

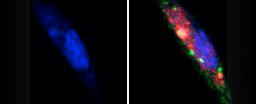

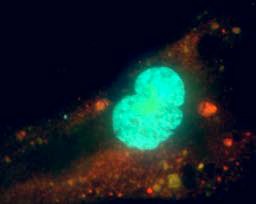

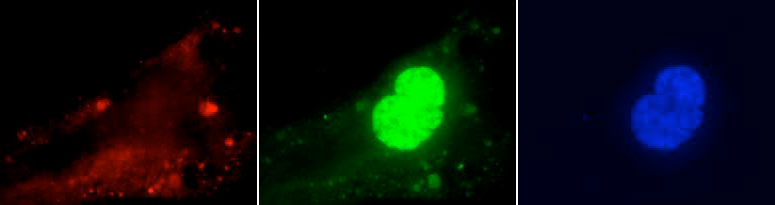


### **G**Untreated RAPA

30,000

**Integrated Density/Cell**

**dsDNA Staining**


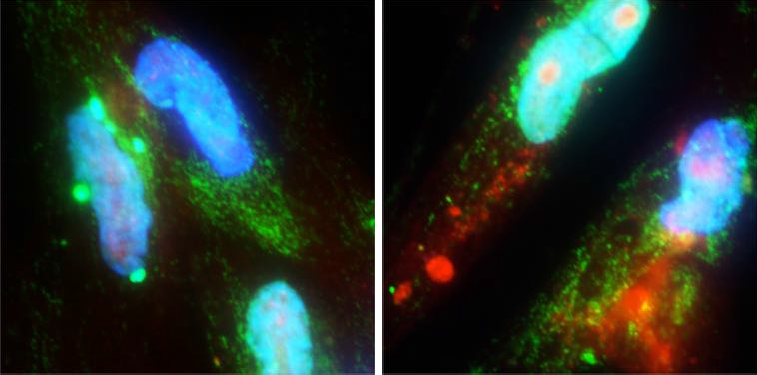

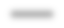

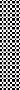

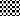

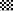


LC3

### *

* 20,000

10,000

LAMP1

### * *

0

Untreated RAPA

***

Nucleus Cytosol

**S1A.** Old MRC5 cells post ﬁxa2on and permeabiliza2on, untreated or treated with DNase1 (500 U/ ml) before staining with an2GdsDNA an2body (red). DAPI, counterstain; scale bar, 100 μm.

**S1B.** Old MCR5 cells liveGstained with MitoTracker Orange (Molecular Probe M7510, 1 mM, 45 min, 37°C), then ﬁxed, permeabilized and stained with an2GdsDNA an2body (green). DAPI, counterstain; scale bar, 20 μm.

**S1C.** Transcript expression of autophagy genes in young and old MRC5 cells assessed by RTGqPCR. **S1D.** IF staining and quan2ta2on of an2GLC3 (red, top panel) and an2GLAMP1 (red, boZom panel) in young and old MRC5 cells, DAPI, counterstain. Quan2ta2on based on 5 random ﬁelds of 10X images; scale bar, 20 μm.

**S1E.** Representa2ve twoGcolor confocal images of an2GLC3 (top panel) and an2GLAMP1 (boZom panel) with PicoGreen (for dsDNA) in MRC5 old cells. Asterisks highlight signal coGlocaliza2on areas; DAPI, counterstain; scale bar, 20 μm.

**S1F.** SAGβgal ac2vity and quan2ta2on in young and old MRC5 cells, treated without or with 10 μM AraGC for 24 h. Scale bar, 50 μm.

**S1G.** IF staining and quan2ta2on of an2GLC3 (red) and an2GdsDNA (green) in old MRC5 cells untreated or treated with rapamycin (RAPA, 100 nm, 24 h), DAPI, counterstain. Scale bar, 20 μm. Signiﬁcance determined by tGtest; p<0.05, *, p<0.01, **, p<0.005, ***, p<0.0001, ****.


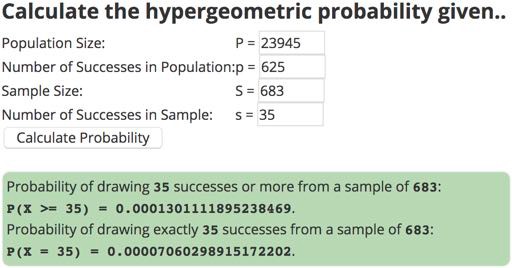

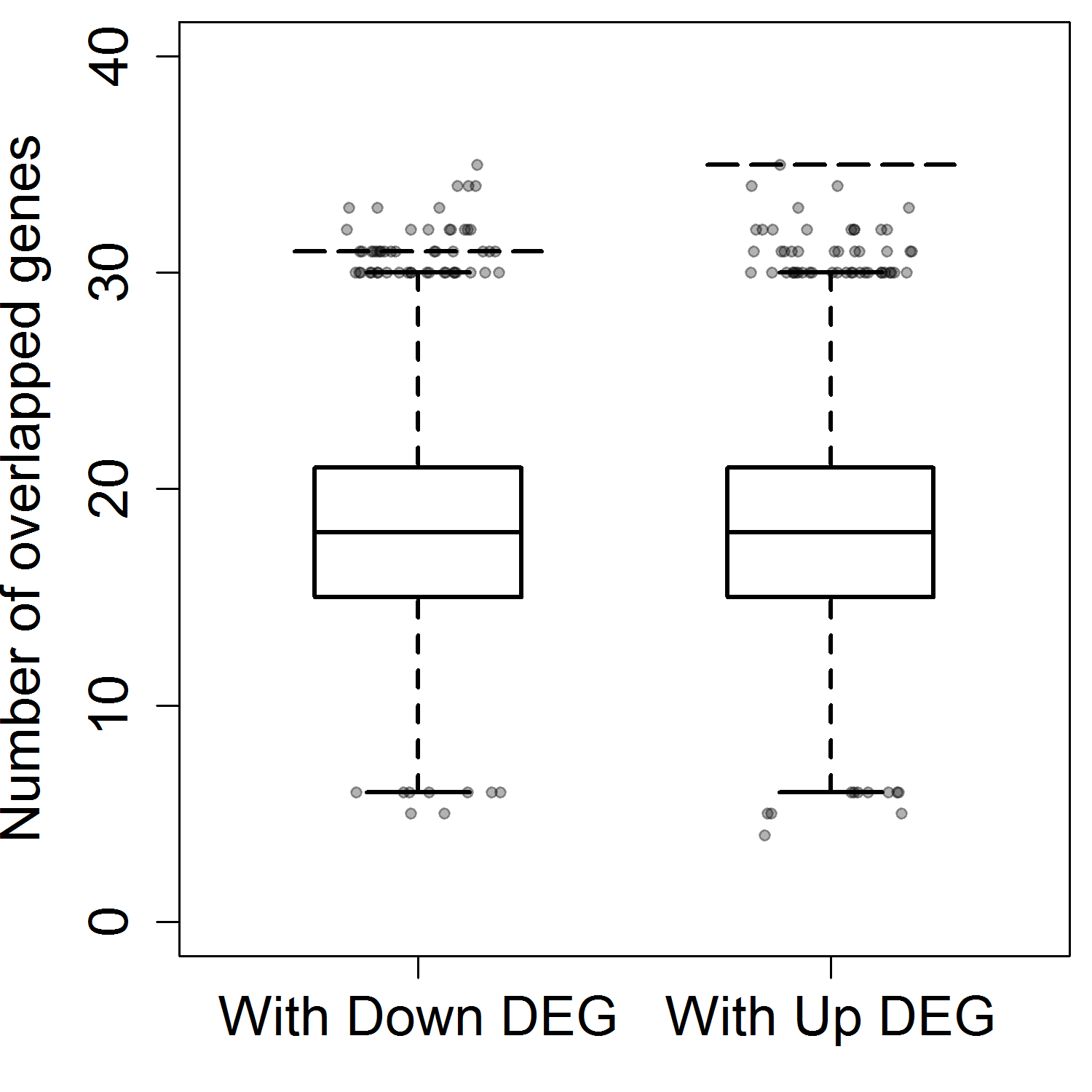

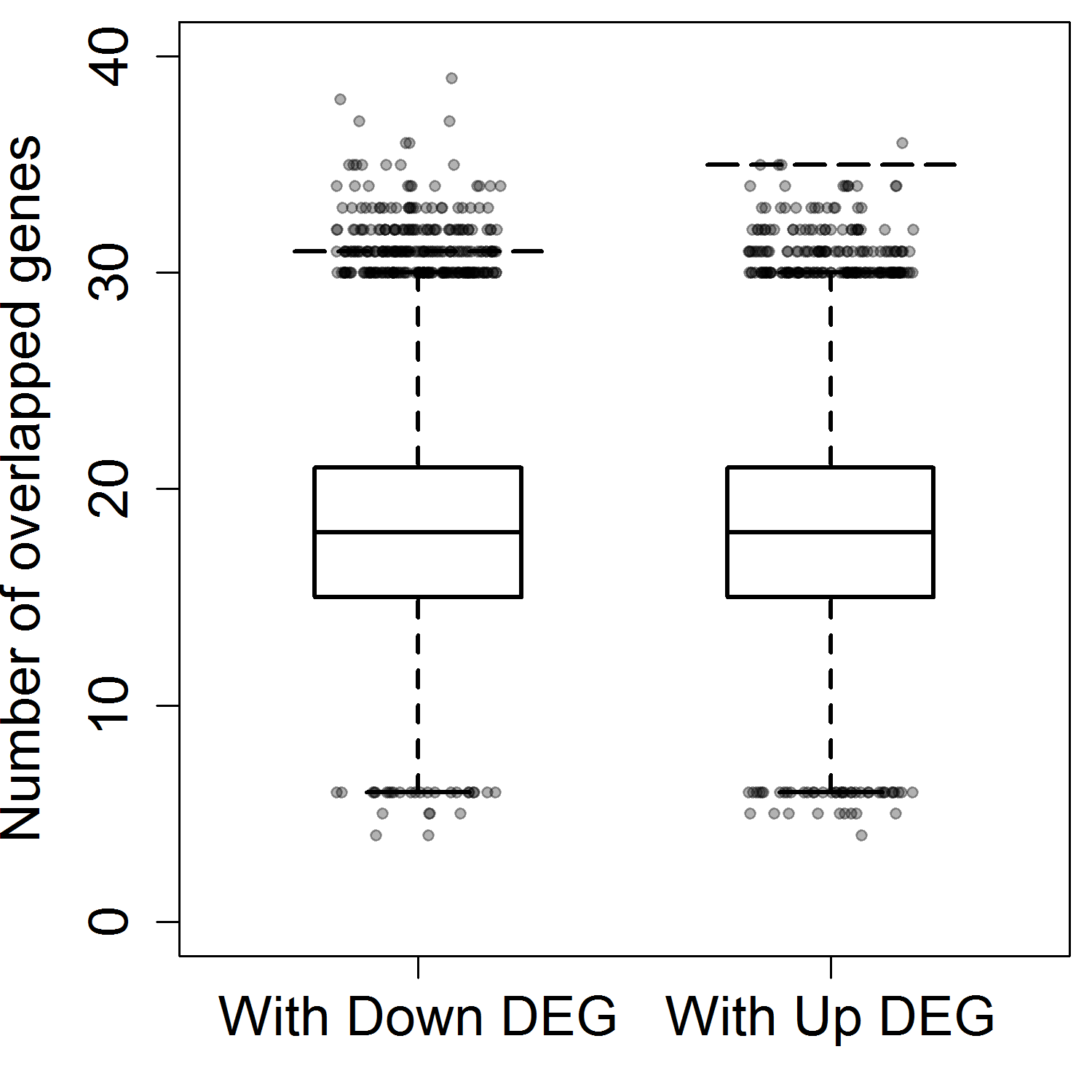


10,000

50,000

3.1eG3 1eG4

8eG5 3.5eG3

**A**

**UP#REGULATED GENES**

***Innate immunity, inﬂamma-on***

CASP1 CCL1 CREM DCTN5 DDX58 FAS GBP1 GBP3 IFI27 IFIT2 IFIT5 IFITM3 IFNAR2 IL33 IL4R IL6 LILRA3 MAP3K8 NFKB2 NLRP3 OLR1 PTPN1 STAT1 STAT2 STAT3 STAT6 TIRAP TLR8

TNFRSF1A

***Growth and diﬀeren-a-on*** C15orf48 CD2AP

CDC37 DUSP6 FGD4 GADD45A GCOM1 GSTT1 HIST2H2BE HK2 MAPK3 SLFN5

***Other func-ons***

ATP13A3

C12orf23 C17orf48 C6orf192 CASS4 CYBRD1 DSP EDN1 FBN2 FRMD3 GPR68 GSTM3 MCOLN2 PLAT PLAU RAB7L1 RBM43 RNF149

**MEAN RATIO GENE NAME**

**B**

1.92

2.73

1.45

1.42

1.85

2.29

1.82

1.50

3.90

3.03

1.69

1.57

1.56

2.91

1.74

2.96

3.33

4.14

1.59

3.67

5.40

1.41

1.67

1.74

1.45

1.67

2.75

2.43

1.33

Caspase 1

C1C mo4f chemokine ligand 1 CAMP Responsive Element Modulator Dynac4n Subunit 5 DExD/H1Box Helicase 58 Fas Cell Surface Death Receptor guanylate binding protein 1 Guanylate Binding Protein 3 Interferon Alpha Inducible Protein 27

Interferon Induced Protein With Tetratricopep4de Repeats 2 Interferon Induced Protein With Tetratricopep4de Repeats 5 Interferon Induced Transmembrane Protein 3 Interferon Alpha And Beta Receptor Subunit 2 Interleukin 33

Interleukin 4 Receptor Interleukin 6

leukocyte immunoglobulin like receptor A3 Mitogen1Ac4vated Protein Kinase Kinase Kinase 8 Nuclear Factor Kappa B Subunit 2 NLR Family Pyrin Domain Containing 3 oxidized low density lipoprotein receptor 1 Protein Tyrosine Phosphatase, Non1Receptor Type 1 Signal Transducer And Ac4vator Of Transcrip4on 1 Signal Transducer And Ac4vator Of Transcrip4on 2 Signal Transducer And Ac4vator Of Transcrip4on 3 Signal Transducer And Ac4vator Of Transcrip4on 6 TIR domain containing adaptor protein Toll Like Receptor 8 TNF Receptor Superfamily Member 1A

2.89

1.63

1.54

2.56

2.70

1.40

2.89

1.91

2.62

2.24

1.40

1.62

Chromosome 15 open reading frame 48

CD2 Associated Protein Cell Division Cycle 37 Dual speciﬁcity phosphatase 6 FYVE, RhoGEF and PH domain containing 4

Growth Arrest And DNA Damage Inducible Alpha GRINL1A complex locus 1 Glutathione S1transferase theta 1 Histone Cluster 2 H2B Family Member E Hexokinase 2 Mitogen1Ac4vated Protein Kinase 3 Schlafen Family Member 5

1.46

1.68

1.96

2.14

2.28

1.37

2.41

2.44

1.82

7.03

1.94

1.82

2.56

3.62

1.58

1.84

2.26

1.43

ATPase 13A3

Transmembrane protein 263 ADP1ribose/CDP1alcohol diphosphatase, manganese dependent Solute carrier family 18 member B1 Cas scaﬀolding protein family member 4 Cytochrome B Reductase 1

Desmoplakin Endothelin 1 Fibrillin 2

FERM domain containing 3 G protein1coupled receptor 68 Glutathione S1transferase mu 3 mucolipin 2 Plasminogen ac4vator, 4ssue type Plasminogen Ac4vator, Urokinase RAB7, member RAS oncogene family1like 1 RNA binding mo4f protein 43 Ring Finger Protein 149

**C**

**D**

| **UP (35)** | **DOWN (31)** |
| --- | --- |
| ABTB2 IFI30 GPER1 HIST1H2BD HIST2H2AA4 GMPR ATF3 HIST2H2BE DPP4 HIST1H4H HIST2H2AC  ANKHD1?EIF4EBP3 TRIM22  ZMAT3 SCD5 IFIT2 SLFN12 FAM46A TRIM38 DUSP6 CREBL2 TRADD GAN PIK3CB BMPR2 DCAF6 MACF1 SAMHD1 GLS B3GNT2 PTPRA SYTL3 SYTL3 PML  RBMS2 | WARS PSMA4 RPL17 PSMA2 PSMA6 MDK GEM IRF2 CCDC109B CEP57 FAM129A AGPAT5 TIMM21 MCM6 MASTL CDC45  C1orf112 EXO1 KNTC1  C21orf58 RFC3 RFC4 ASF1B BRI3BP POLE2 AKAP7 MCM10 HIST1H4C PRIM1 ATAD5 HIST2H2AC |

**DOWN%REGULATED GENES MEAN RATIO GENE NAME**

***Innate immunity, inﬂamma-on***

HMGB1

HMGB2 HMGB3

***Growth and diﬀeren-a-on***

AXL BRIP1 CD24 CKB HIRA PTGER2 SEMA3D VCAN

***Other func-ons*** ATP1B1 CCDC109B GEM

PTMA SLC1A3

0.59

0.47

0.52

High mobility group box 1

High mobility group box 2 High mobility group box 3

0.59

0.64

0.41

0.57

0.62

0.64

0.49

0.52

AXL receptor tyrosine kinase

BRCA1 interacting protein C-terminal helicase 1 CD24 molecule

Creatine kinase B

Histone cell cycle regulator

Prostaglandin E receptor 2 (subtype EP2) Semaphorin 3D

Versican

0.75

0.43

0.58

0.59

0.37

ATPase Na+/K+ transporting subunit beta 1

Mitochondrial calcium uniporter dominant negative beta subunit GTP binding protein overexpressed in skeletal muscle Prothymosin, alpha

Solute carrier family 1 member 3

**G STING Genes**


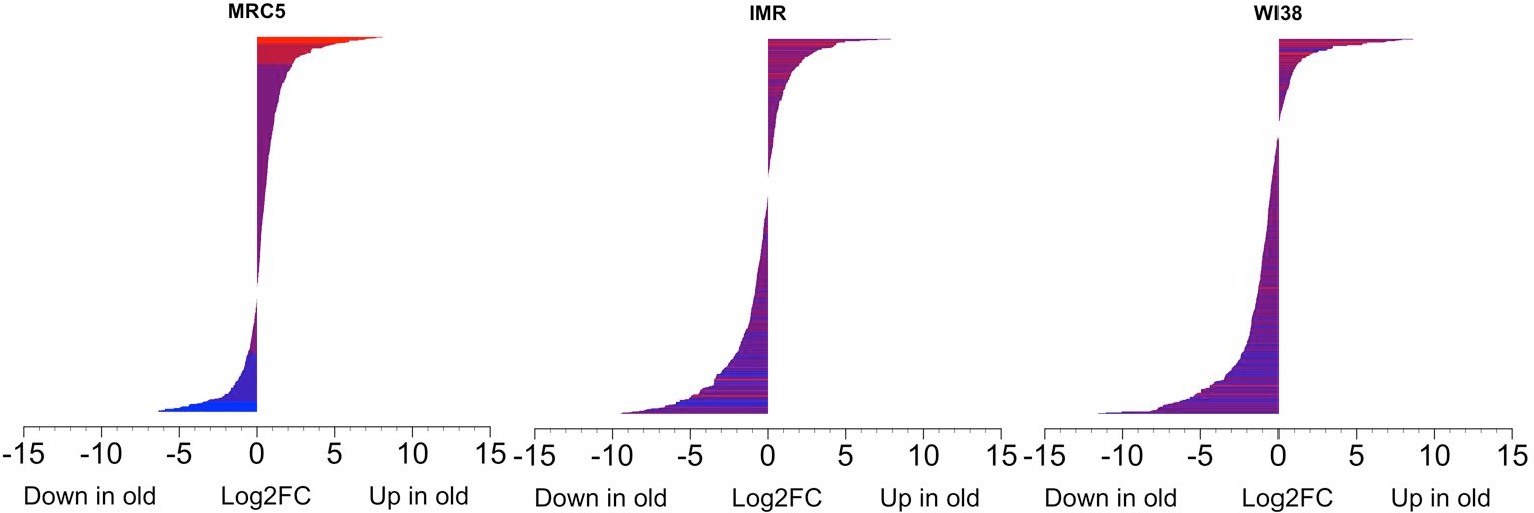


**E**

MRC5

IMR90

WI38

|  |
| --- |
|  |
|  |
| IL1A  IGHE ZDHHC1 PLA2G6 |
| F3 IKBKB |
| DDX58 HRH4 NLRC3 |
| TSLP SAMHD1 DTX4 |
| TRIM32 BAX |
| IL4R  MAVS MT<ND1 SCP2 TRAF3 TRIM56 STAT6 PPM1A HRH1 UBE2D1 NEXN MRI1 TREX1 TMEM173 TRIM21 TBK1 TFG IRF3 WASL |
| DDX41  IFI16 TRAF6 KITLG TICAM1 UBE2D3 ISG20 ULK1 PRKDC TPT1 MB21D1 XRCC6 XRCC5 SSR2 TRAF2 NFKBIA CCL5 SLC6A4 FCER2 POMC IL10 |

1. **H** 4

mRNA (% B2M)

| **Enriched in Old** | **FDR q7val** |
| --- | --- |
| HALLMARK_TNFA_SIGNALING_VIA_NFKB HALLMARK_P53_PATHWAY HALLMARK_INFLAMMATORY_RESPONSE HALLMARK_KRAS_SIGNALING_UP HALLMARK_HEME_METABOLISM HALLMARK_INTERFERON_ALPHA_RESPONSE HALLMARK_INTERFERON_GAMMA_RESPONSE HALLMARK_APOPTOSIS HALLMARK_MYOGENESIS HALLMARK_KRAS_SIGNALING_DN HALLMARK_IL2_STAT5_SIGNALING HALLMARK_HYPOXIA HALLMARK_ESTROGEN_RESPONSE_EARLY HALLMARK_UV_RESPONSE_UP HALLMARK_NOTCH_SIGNALING HALLMARK_IL6_JAK_STAT3_SIGNALING HALLMARK_COAGULATION HALLMARK_PEROXISOME HALLMARK_XENOBIOTIC_METABOLISM HALLMARK_ADIPOGENESIS | 0.00000  0.00000  0.00000  0.01031  0.01080  0.01064  0.01337  0.01204  0.01797  0.02107  0.04706  0.04957  0.07067  0.07519  0.08591  0.11277  0.17488  0.16768  0.22617  0.24059 |
| HALLMARK_EPITHELIAL_MESENCHYMAL_TRANSITION | 0.33919 |
| HALLMARK_ANGIOGENESIS | 0.34802 |
| HALLMARK_TGF_BETA_SIGNALING | 0.39715 |
| HALLMARK_UV_RESPONSE_DN | 0.91599 |
| HALLMARK_PROTEIN_SECRETION | 0.91273 |

3

2

1

**siRNA Knockdown**

Young


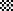


****


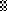

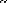

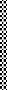

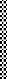

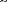


****

****

Old

0 **** * ***

CGAS STING TBK1

**I Old cells_siSTING/siNEG**

*

2.5

2.0

**

1.5

1.0

*

*

0.5

0.0

**Fold change (mRNA)**

**S2A.** Classiﬁed list of 59 signiﬁcant upG or downGregulated genes in old vs. young MRC5 cells among 413 innate and inﬂammatory genes in the NanoString panel.

**S2B.** Type I IFN genes that overlap with DEGs in old cells, 35 upGregulated and 31 downGregulated. **S2C.** Hypergeometric probability test for upGregulated overlapping genes in S2B.

**S2D.** Boxplots showing permuta2on test of overlapping genes in S2B by random sampling (lei = 10,000, right = 50,000 samples). Red horizontal lines represent actual frequencies above predicted means in upGor downGregulated genes.

**S2E.** Horizontal bar plots of overlapping genes in Fig. S2B based on log2 fold change expression in old vs. young in cell lines indicated. Genes follow the order from high to low fold change in MRC5. **S2F.** Enriched GSEAGranked gene sets in old cells across the 3 cell lines of IMR90, MRC5 and WI38, FDR<0.25 highlighted.

**S2G.** List of STINGGrelated genes with DEGs in old cells highlighted.

**S2H.** Assessment of siRNA knockdown eﬃciency for *cGAS*, *STING* and *TBK1* in young and old MRC5 cells by RTGqPCR.

**S2I.** Fold change mRNA expression (siSTING/siNEG) of SASP factors in oldMRC5 cells by RTGqPCR. Signiﬁcance determined by tGtest; p<0.05, *, p<0.01, **, p<0.005, ***, p<0.0001, ****; if not indicated.

**A** 40,000


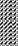

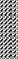

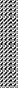


**Integrated Density/Cell**

No pIRF3

γ**H2AX C**

30,000

20,000

10,000

***

* *

### A549

0

**B** H1 H2 AT1 AT2 AT3 PS1 PS2


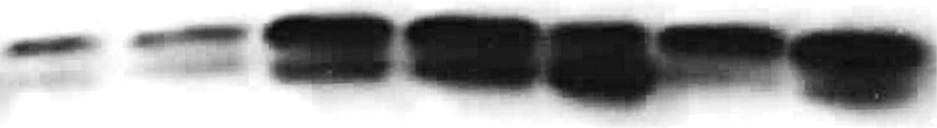

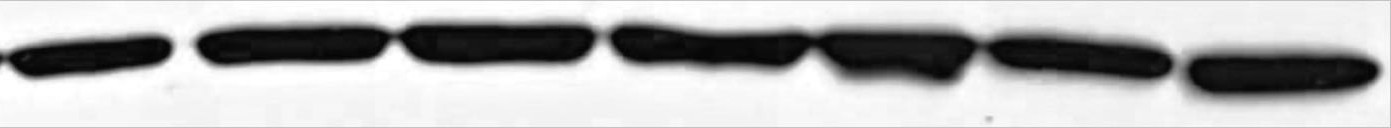

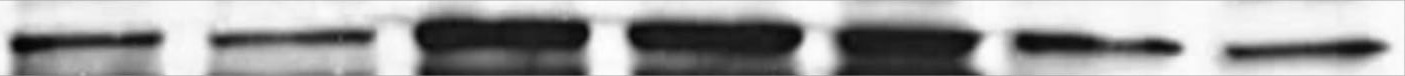

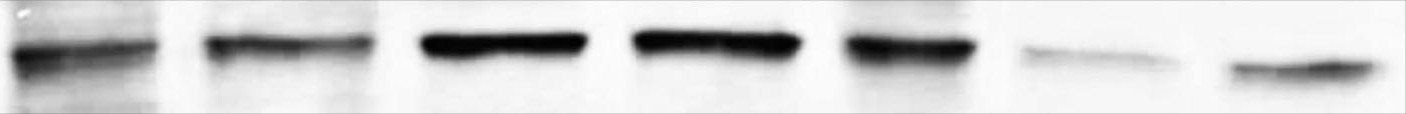


**D** 0.8

pSTING STING

TBK1 IRF3 β&ACTIN

### A549

H2

0.6

**mRNA (% GAPDH)**

0.4

**STING**

siNEG siSTING


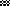

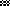

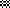

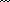

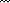

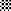


pIRF3

### F5

0.2

0.0

H1 H2 AT1 AT3 PS1 PS2


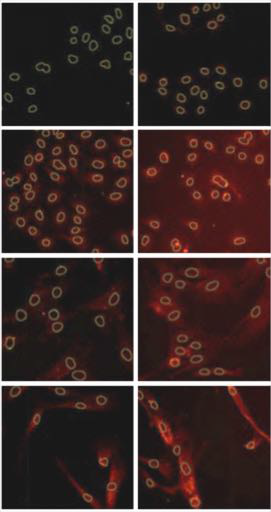


No DNA

DNA

*

*

*

*

*

*

*

*

*

*

*

*

*

*

*

*

**S3A.** Quan2ta2on of γGH2AX IF staining in healthy (H), ataxia (AT) and progeria (PS) skin ﬁbroblasts. Numbers represent diﬀerent ﬁbroblasts of each phenotype (Coriell catalog no. listed in Experimental Procedures). Signiﬁcance by 1Gway ANOVA between groups, p<0.0001; and that of AT2, PS2 and PS3 vs. H1 or H2 by Tukey’s test as indicated.

**S3B.** Immunoblot showing DNA sensing mediators in H, AT and PS cells, double bands in total STING are visible in disease cells, βGACTIN, loading control.

**S3C.** A549 cells (human epithelial) and human ﬁbroblasts from pa2ents are transfected with or without GAPDH DNA generated by PCR or ISD (interferon s2mulatory DNA) fragments respec2vely (both 4 μg/ml, 4 h), using TransITGLT1 transfec2on reagent (Mirus Bio, TransIT:DNA = 3:1). Top panel shows background staining with secondary an2Grabbit an2body (AF568, red) alone. Yellow nuclear boundaries are based on DAPI staining. Asterisks highlight nuclear pIRF3 staining; scale

bar, 20 μm.

**S3D.** STING knockdown eﬃciency in H, AT and PS cells validated by RTGqPCR.

1. **SASP Factors**

50


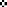

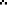

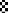

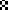

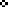

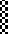

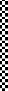

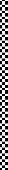

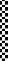

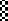

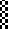

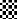


siNEG

**B**

250

**mRNA (% GAPDH)**

**DNASE2A**

siDNASE2A

40

**Fold change**

30

20

10

***

*

***

***

200

150

100

50

0


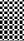


Young Old

eGFP DNASE2A


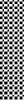

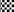


| **** | | | | |
| --- | --- | --- | --- | --- |
| **** | | |  |  |
|  |  |  |  |  |

***

0

*** **

#### Young Old

**S4A.** Fold change (siDNASE2A/siNEG) of transcript expression of SASP factors in young and old MRC5 cells with *DNASE2A* knocked down by transfected siRNAs; *siNEG*, nonGtarge2ng control. Expression values assessed by RTGqPCR, signiﬁcance rela2ve to *siNEG* values.

**S4B.** Assessment of human *DNASE2A* expression in young and old MRC5 cells aier transduc2on with *DNASE2A* ORF or control eGFP by RTGqPCR. Signiﬁcance based on expression in eGFP controls. *DNASE2A* levels in eGFP controls readily detectable but barely visible in scale shown.

Signiﬁcance by tGtest, *, p<0.05; **, p<0.01; ***, p<0.005; ****, p<0.001.

**A** 100

80

60


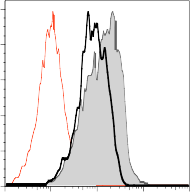


**% of Max**

1. **SASP factors_MLFs**

15

**-/- / +/+ Fold Change**

**D** +/+ +/+ +/+ &/& &/& &/&

### p21 p53

G +/+

40 10

20 G G/G

0 5

****

****

### β&ACTIN

100 101 102 103 10

**Ki67**

Ki67

1 ***

0

**

** ** ****


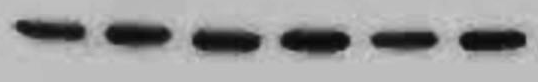

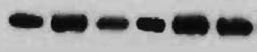

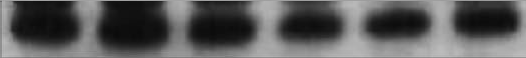


***

1. Kidney Heart


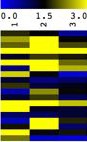

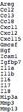


*

*


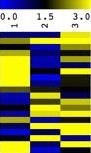

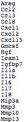


*

*

*

**E**

+/+


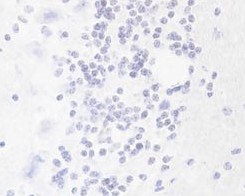

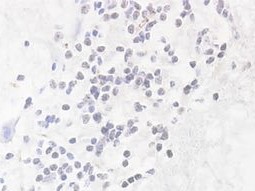

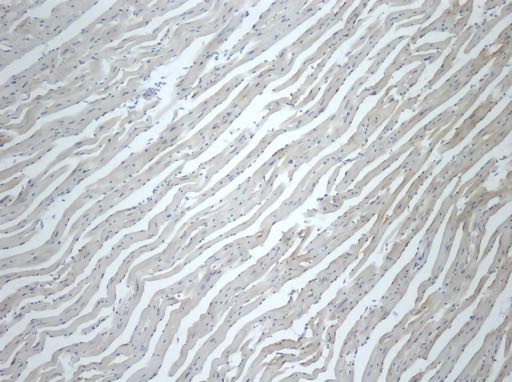

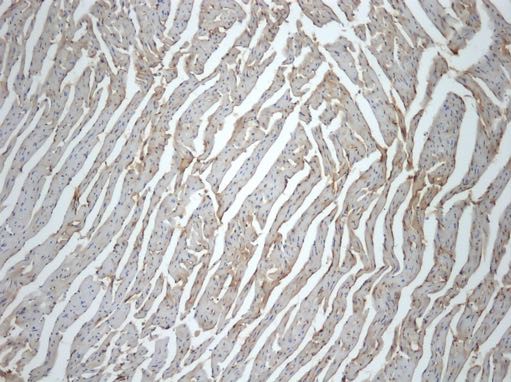


Brain

Heart

>/>

**S5A.** Cell prolifera2on of *Dnase2a*+/+ and *Dnase2a*G/G MLFs assessed by Ki67 staining using ﬂow cytometry; *Dnase2a*+/+, shaded histogram, *Dnase2a*G/G, open histogram; isotype control, red open histogram.

**S5B.** Fold change of transcript expressions of SASP factors in *Dnase2a*G/G vs. *Dnase2a*+/+

### MLFs by RTGqPCR. Representa2ve of 2 independent experiments shown.

**S5C.** Heat map showing fold change of gene expression of SASP factors in *Dnase2a*G/G to *Dnase2a*+/+ kidney and heart 2ssues from 3 pairs of age and sexGmatched liZermates. Asterisks indicate signiﬁcant genes.

**S5D.** Immunoblot of p21 and p53 in kidney 2ssues from 3 pairs of age and sexGmatched

*Dnase2a*+/+ and *Dnase2a*G/G liZermates.

### **S5E.** Immunohistochemical staining of p16 in brain and heart 2ssues from *Dnase2a*+/+ and *Dnase2a*G/G mice by DAB chromogen. Posi2ve signals appear in brown, representa2ve of 2 independent experiments, scale bar, 200 μm.

Signiﬁcance by tGtest if not indicated.

**Supplementary Experimental Procedures**

#### Mice

Inducible *Dnase2a* KO mouse was a gii from Dr. Shigekazu Nagata (Kyoto University, Japan). WT and KO liZermates were injected *i.p.* with 1.5 μg/weight (g) of poly I:C three 2mes at 12G16 weeks of age to induce dele2on of *Dnase2a*. Mice were housed in a speciﬁc pathogenGfree facility at MassachuseZs General Hospital. The MGH SubcommiZee on Research and Animal Care approved all protocols and procedures for animal studies in accordance with the ins2tu2onal animal ethics guidelines. Mouse 2ssues were ﬁxed with 4% paraformaldehyde (PFA) overnight, dehydrated by 30% sucrose and frozen in OCT medium for cryoGsec2oning.

#### Reagents

AraGC, baﬁlomycin A1 and rapamycin were from SigmaGAldrich (St. Louis, MO); SA βGgal Staining Kit from Cell Signaling (Danvers, MA); TUNEL ApoGGreen detec2on reac2on mixture from Biomake (Houston, TX).

#### Microscopy

IF (10X, 20X) or brightGﬁeld images (5X, 10X) were captured using Zeiss Axio Imager M2 upright microscope, Axiocam 506 and ZEN Blue soiware or by Axio Scan.Z1 with ZEN scan soiware.

#### NanoString proﬁling and data normalizaIon

Lysates in RLT buﬀer were hybridized for 12G24 hours with custom nCounter Gene Expression CodeSets. Hybridized RNA transcripts in a mul2plex reac2on were counted by the Nanostring nCounter system (Nanostring; SeaZle, WA). Results for mul2ple probes detec2ng the same gene were averaged and rounded to the nearest integer. Four reference genes (PHLDA1, SPRY2, SEMA3A and C9orf30) were selected based on uniform expression and used as “housekeeping” genes. Signiﬁcant genes diﬀeren2ally expressed were determined by NanoStringDiﬀ R package (version 1.4.0)1, using a Benjamin and Hochberg adjusted p value (FDR) threshold of 0.01.

#### RNA sequencing and analysis

RNA was isolated with RNeasy→ Plus Mini Kit (Qiagen, MD). 1 ng RNA was used as template to generate fullGlength cDNA and sequencing libraries using the SmartGSeq2 protocol as previously described2.

Libraries were sequenced on a NextSeq 500 (Illumina) to an average depth of 12.4 million pairedGend reads of length 38 bases each. Reads were mapped to the Gencode_v19 human transcriptome using Bow2e 23 and expressions of all genes quan2ﬁed using RSEM4 to yield an expression matrix (genes x samples) of inferred gene counts. Diﬀeren2al expression was calculated with EBSeq version 1.10.05, using the EBTest func2on with ten itera2ons following normaliza2on using the MedianNorm func2on. Three rows were removed from the gene count table prior to analysis; ENSG00000225840, a Y chromosome rRNA pseudogene with high levels of aberrant mapping, and two mitochondrial rRNA genes (MTGRNR1 and MTGRNR2).

Gene ontology func2onal enrichment was assessed using Overrepresenta2on Enrichment Analysis on the WebGestalt online tool6, comparing signiﬁcantly diﬀeren2ally expressed genes (posterior probability of diﬀeren2al expression >0.95) against reference gene set containing all genes with nonGzero transcript levels. Gene set enrichment analysis7 was run on the GenePaZern pla}orm8. 1000 geneGset permuta2ons were used, working from the MSigDB Hallmark gene set collec2on9.

#### Knockdown experiments

Cells were transfected with 150 nM of siGENOME pool siRNA (Dharmacon, CO) targe2ng *cGAS*, *STING, TBK1, DNASE2A* or nonGtarge2ng control, using Lipofectamine™ RNAiMAX (Life Technologies).

Knockdown eﬃciency was conﬁrmed by RTGqPCR.

#### ImmunobloOng

Cells were lysed in RIPA buﬀer (Boston Bioproducts, Worcester, MA) supplemented with Complete mini protease inhibitor cocktails (Roche, Indianapolis, IN) and protein concentra2on determined by BioGRad protein assay (Hercules, CA). 10G30 μg of protein was separated by SDSGPAGE in 10 or 12% miniG PROTEAN® precast gel (BioGRad) and transferred to PVDF membrane. Membrane was then blocked with 5% nonGfat dry milk and immunobloZed with the following an2bodies:

STING Cell Signaling #13647 1:1000

TBK1 Cell Signaling #3504 1:1000

pTBK1 Cell Signaling #5483 1:1000

IRF3 Cell Signaling #4302 1:1000

HPG1β Cell Signaling #2613 1:1000

JAK1 Cell Signaling #3332 1:1000

STAT1 Cell Signaling #9172 1:1000

pSTAT1 Cell Signaling #9167 1:1000

βGACTIN Abcam ab6276 1:10,000

| Mouse p16 | Abcam ab211542 | 1:1000 |
| --- | --- | --- |
| Mouse p21 | Santa Cruz scG6246 | 1:1000 |
| Mouse p53 | Santa Cruz scG98 | 1:1000 |

#### Cell proliferaIon

Duplicate wells of 100,000 cells were plated in 6Gwell plates and counted daily for 5 days by trypan blue to exclude dead cells. Cells were trypsinized to split and reGplate upon 80G90% conﬂuence (on day 3 or 4) to allow suﬃcient growth space. Both split wells were included in ﬁnal count. Alterna2vely, cells were stained with PE conjugated KiG67 an2body (Biolegend 652404) and analyzed by ﬂow cytometry.

To assess cell growth over longer dura2on, triplicate wells were plated and counted at conﬂuence. Equal numbers of cells were then serially plated at each split. Cell growth then calculated as cumula2ve PD at each passage.

#### DNA digesIon assay

Two million MRC5 young and old cells were pelleted and lysed in 80 μl of 20 mM TrisGHCl with protease inhibitors, pH=7.5. Diﬀerent volumes of lysates were incubated with 50 μg of calf thymus DNA in a total volume of 45 μl of 25mM of sodium acetate (pH=4.7) for 15 min at room temperature. Digested DNA products were loaded onto 0.7% agarose gel and visualized by ethidium bromide. Dilu2ons of porcine DNASE2 (Sigma D4138) were used as posi2ve control.

#### RealQIme RTQqPCR

0.25G1 μg of total RNA was reverseGtranscribed with High Capacity cDNA Reverse Transcrip2on kit (Applied Biosystems). Quan2ta2ve PCR was then performed using LightCyler→ 480 SYBR Master I reagents (Roche) on LightCyler→ 96 instrument. Transcript levels were normalized to *B2M* or *GAPDH*. Primer pairs used are listed in table below.

| **HUMAN PRIMERS** | **5'Q3' SEQUENCE** |
| --- | --- |
| *ATG5*-F | AAAGATGTGCTTCGAGATGTGT |
| *ATG5*-R | CACTTTGTCAGTTACCAACGTCA |
| *BECN1*-F | CCATGCAGGTGAGCTTCGT |
| *BECN1*-R | GAATCTGCGAGAGACACCATC |
| *P62*-F | TGCCCAGACTACGACTTGTG |
| *P62*-R | AGTGTCCGTGTTTCACCTTCC |
| *PTEN*-F | TGGATTCGACTTAGACTTGACCT |
| *PTEN*-R | TGGCGGTGTCATAATGTCTTTC |
| *cGAS*-F | TAACCCTGGCTTTGGAATCAAAA |
| *cGAS*-R | TGGGTACAAGGTAAAATGGCTTT |
| *STING*-F | GGTCACCGCTCCAAATATGTAG |
| *STING*-R | CAGTAGTCCAAGTTCGTGCGA |
| *TBK1*-F | AGCGGCAGAGTTAGGTGAAA |
| *TBK1*-R | TGAGTGCCTTCTTGATGTGC |
| *MX1*-F | GTTTCCGAAGTGGACATCGCA |
| *MX1*-R | CTGCACAGGTTGTTCTCAGC |
| *IFIT1*-F | TTGATGACGATGAAATGCCTGA |
| *IFIT1*-R | CAGGTCACCAGACTCCTCAC |
| *IL6*-F | AAATTCGGTACATCCTCGACGG |
| *IL6*-R | GGAAGGTTCAGGTTGTTTTCTGC |
| *CXCL10*-F | CCAAGTGCTGCCGTCATTTTC |
| *CXCL10*-R | GGCTCGCAGGGATGATTTCAA |
| *DNASE2A*-F | TCGCCTTCCTGCTCTACAAT |
| *DNASE2A*-R | CCCATCTTCGAGAACTGAGC |
| *AREG*-F | GTGGTGCTGTCGCTCTTGATA |
| *AREG*-R | CCCCAGAAAATGGTTCACGCT |
| *GMCSF*-F | TTCTGCTTGTCATCCCCTTT |
| *GMCSF*-R | CTTCTGCCATGCCTGTATCA |
| *IGFBP7*-F | CGAGCAAGGTCCTTCCATAGT |
| *IGFBP7*-R | GGTGTCGGGATTCCGATGAC |
| *MMP3*-F | AGTCTTCCAATCCTACTGTTGCT |
| *MMP3*-R | TCCCCGTCACCTCCAATCC |
| *MMP10*-F | TGCTCTGCCTATCCTCTGAGT |
| *MMP10*-R | TCACATCCTTTTCGAGGTTGTAG |
| *MMP13*-F | ACTGAGAGGCTCCGAGAAATG |
| *MMP13*-R | GAACCCCGCATCTTGGCTT |
| *P16*-F | GGGTTTTCGTGGTTCACATCC |
| *P16*-R | CTAGACGCTGGCTCCTCAGTA |
| *P21*-F | TGTCCGTCAGAACCCATGC |
| *P21*-R | AAAGTCGAAGTTCCATCGCTC |
| *B2M*-F | CTCCGTGGCCTTAGCTGTG |
| *B2M*-R | TTTGGAGTACGCTGGATAGCCT |
| *GAPDH*-F | ACAACTTTGGTATCGTGGAAGG |
| *GAPDH*-R | GCCATCACGCCACAGTTTC |

| **MOUSE PRIMERS** | **5'Q3' SEQUENCE** |
| --- | --- |
| *p16*GF | GAACTCTTTCGGTCGTACCC |
| *p16*GR | CGAATCTGCACCGTAGTTGA |
| *p21*GF | CCTGGTGATGTCCGACCTG |
| *p21*GR | CCATGAGCGCATCGCAATC |
| *Ccl8*GF | TCTACGCAGTGCTTCTTTGCC |
| *Ccl8*GR | AAGGGGGATCTTCAGCTTTAGTA |
| *Cxcl2*GF | GCGCCCAGACAGAAGTCATAG |
| *Cxcl2*GR | AGCCTTGCCTTTGTTCAGTATC |
| *Il1b*GF | CCAGCTTCAAATCTCACAGCAG |
| *Il1b*GR | CTTCTTTGGGTATTGCTTGGGATC |
| *Il8*GF | TCGAGACCATTTACTGCAACAG |
| *Il8*GR | CATTGCCGGTGGAAATTCCTT |
| *Dnase2*GF | GCTCAGCTGGGGACTCTAC |
| *Dnase2*GR | GGTCTGGCCGAAGGTTTGA |
| *Gapdh*GF | AGGTCGGTGTGAACGGATTTG |
| *Gapdh*GR | TGTAGACCATGTAGTTGAGGTCA |

#### StaIsIcal analyses

Sta2s2cal analyses were performed using GraphPad PRISM 4 or as described. Values were expressed as mean±sem. Samples were analyzed using Student’s tGtest or as indicated, with p<0.05 deemed sta2s2cally signiﬁcant and denoted by *; p<0.05, *; p<0.01, **; p<0.005, ***; p<0.0001, ****.

#### Supplementary references

1. Wang, H. *et al.* NanoStringDiﬀ: a novel sta2s2cal method for diﬀeren2al expression analysis based on NanoString nCounter data. *Nucleic Acids Res.* gkw677 (2016).
2. Picelli, S. *et al.* FullGlength RNAGseq from single cells using SmartGseq2. *Nat. Protoc.* **9,** 171–181 (2014).
3. Langmead, B. & Salzberg, S. L. Fast gappedGread alignment with Bow2e 2. *Nat. Methods* **9,** 357–359 (2012).
4. Li, B. & Dewey, C. N. RSEM: accurate transcript quan2ﬁca2on from RNAGSeq data with or without a reference genome. *BMC BioinformaIcs* **12,** 1–16 (2011).
5. Leng, N. *et al.* EBSeq: an empirical Bayes hierarchical model for inference in RNAGseq experiments.

*BioinformaIcs* **29,** 1035–1043 (2013).

1. Wang, J., Duncan, D., Shi, Z. & Zhang, B. WEBGbased GEne SeT AnaLysis Toolkit (WebGestalt): update 2013. *Nucleic Acids Res.* **41,** W77–W83 (2013).
2. Subramanian, A. *et al.* Gene set enrichment analysis: A knowledgeGbased approach for interpre2ng genomeGwide expression proﬁles. *Proc. Natl. Acad. Sci.* **102,** 15545–15550 (2005).
3. Michael Reich *et al.* GenePaZern 2.0. *Nat. Genet.* **38,** 500–501 (2006).
4. Liberzon, A. *et al.* The Molecular Signatures Database Hallmark Gene Set Collec2on. *Cell Syst.* **1,** 417– 425 (2015).
